# Supplementary figures and images for: Urban environmental and population factors as determinants of COVID-19 severity: A spatially-resolved probabilistic modeling approach
Source: PLOS Digit Health. 2025 Jul 18;4(7):e0000921. doi: 10.1371/journal.pdig.0000921 (PMC12274012; doi:10.1371/journal.pdig.0000921)

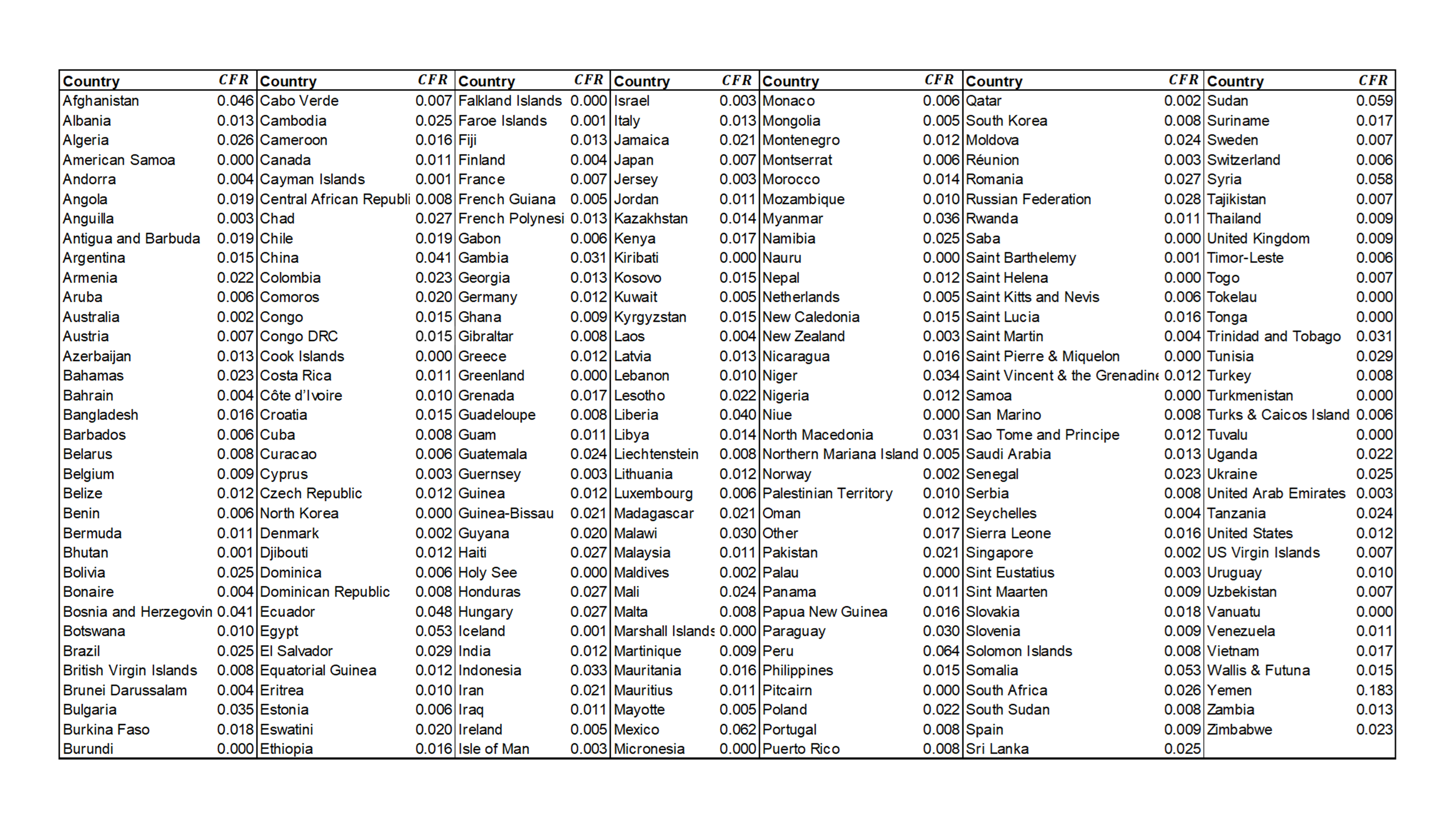

Supplement: S1 Table — (TIF) [file pdig.0000921.s001.TIF]

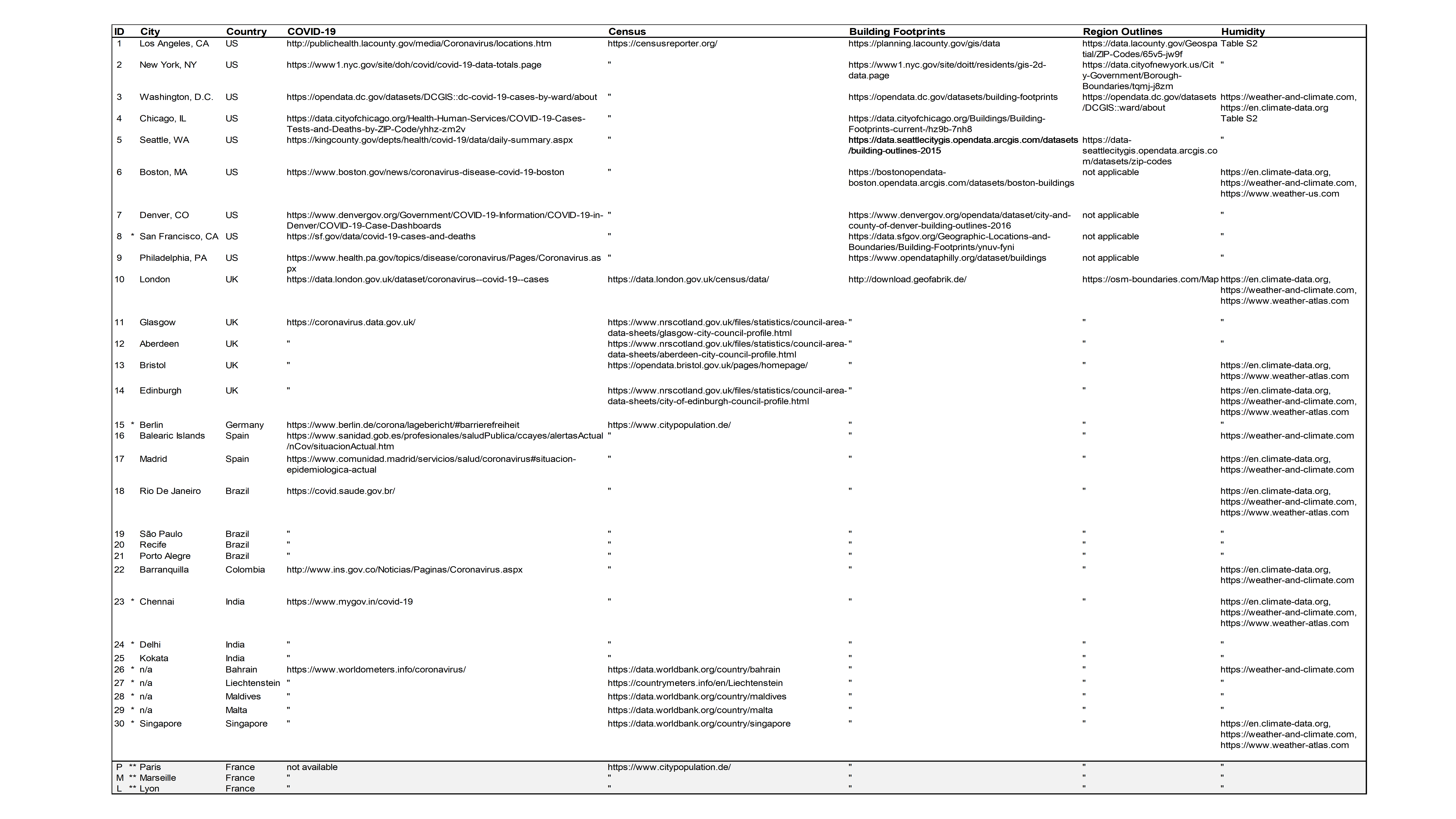

Supplement: S2 Table — Multiple sources for humidity were used to obtain average values presented in S5 Table. (TIF) [file pdig.0000921.s002.TIF]

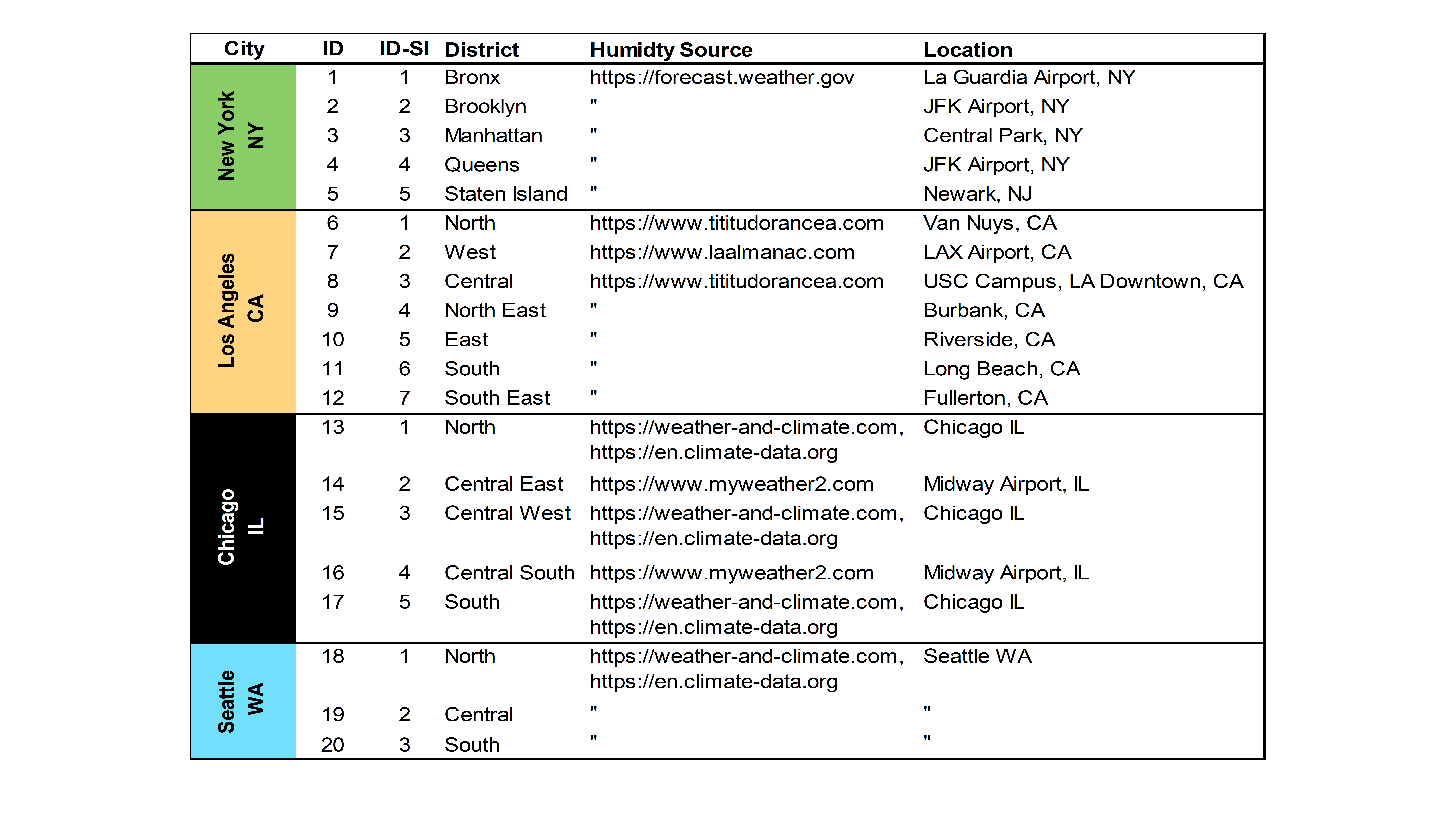

Supplement: S3 Table — All sources for each location were used to derive humidity values in S4 Table. (TIF) [file pdig.0000921.s003.TIF]

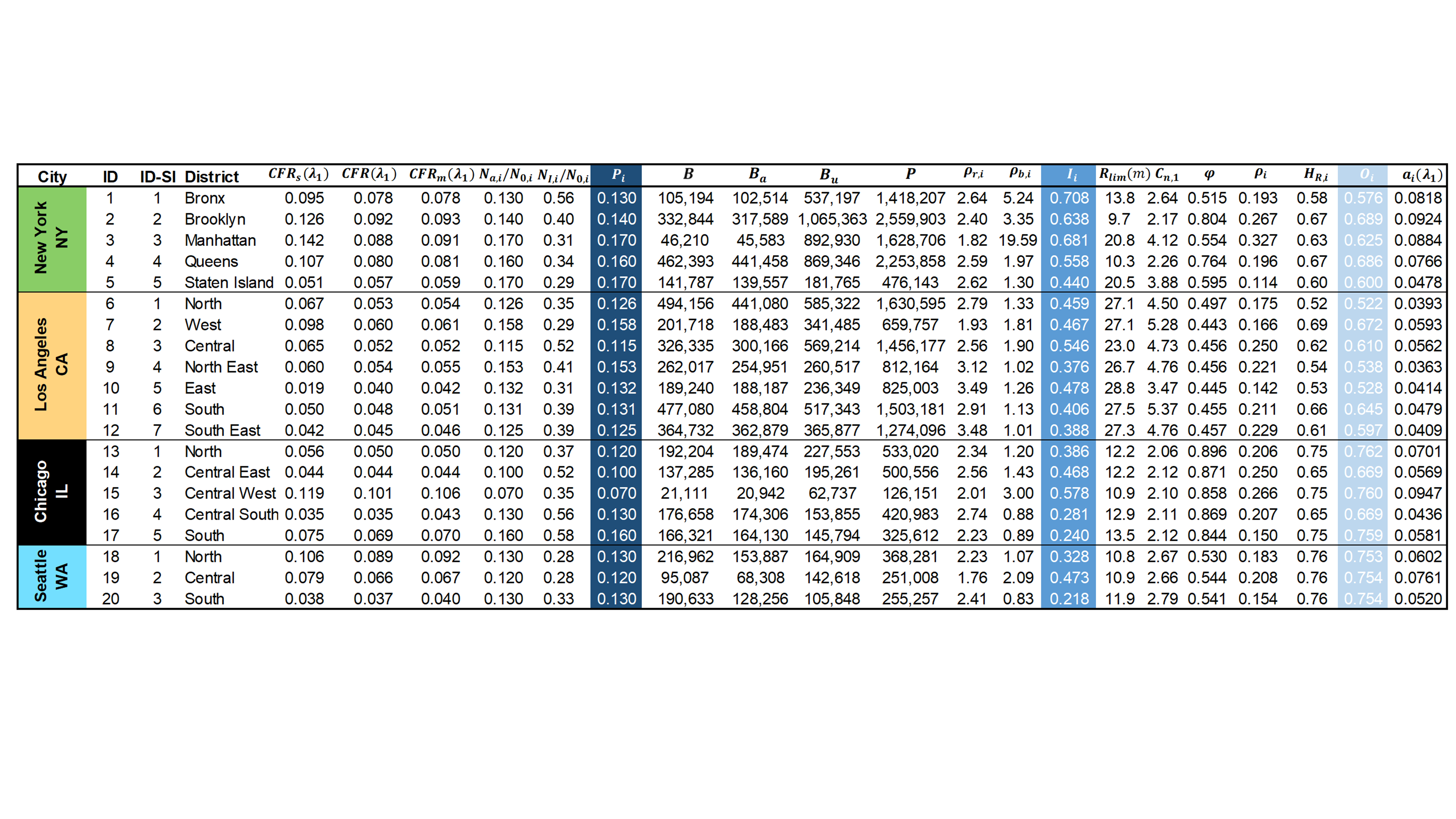

Supplement: S4 Table — District boundaries are defined using boundaries as shown in S1 Fig. (TIF) [file pdig.0000921.s004.TIF]

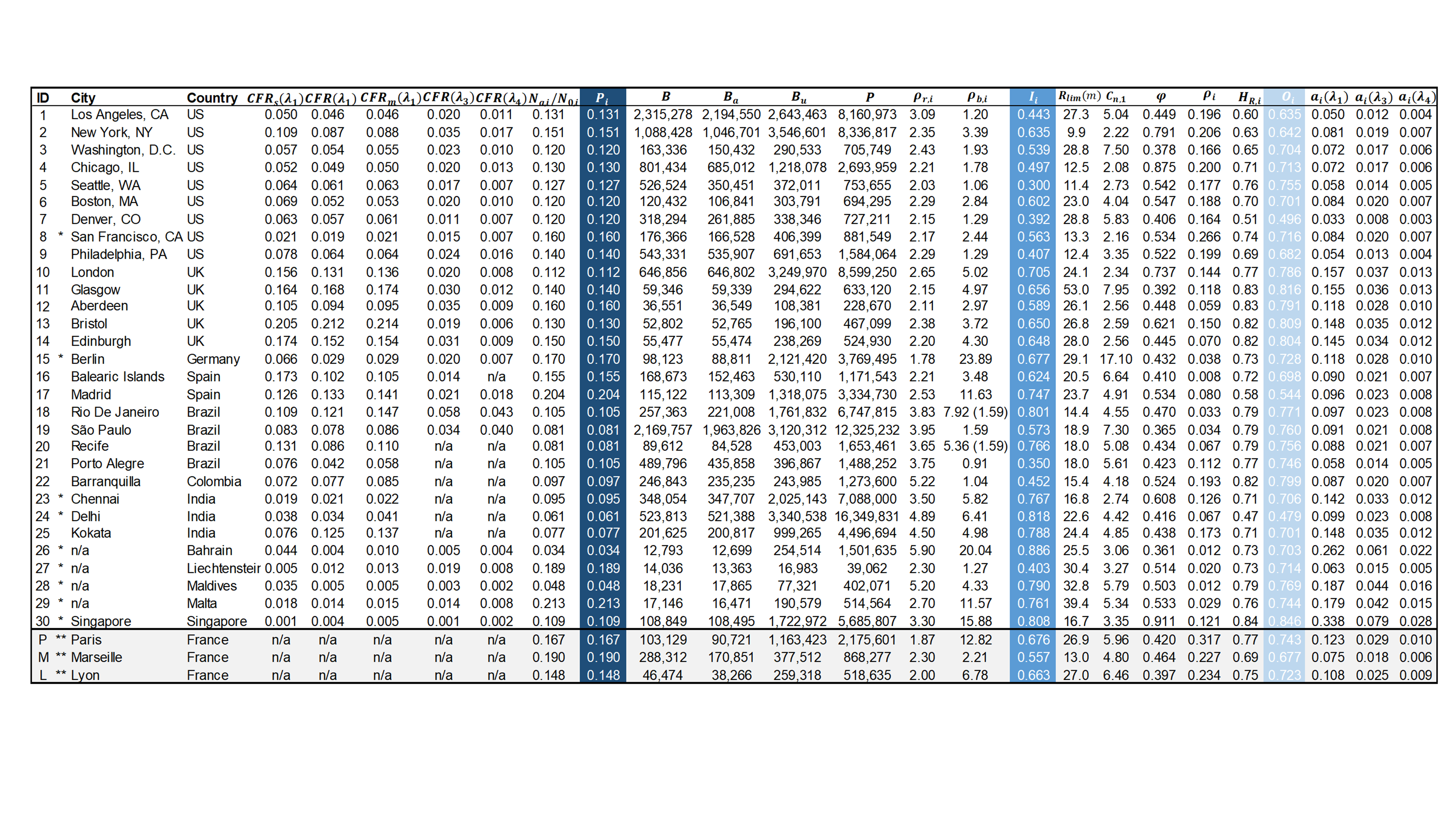

Supplement: S5 Table — *Outliers in CFR predictions – cities with CFR values close to intrinsic CFR, a0. **Cities with no CFR estimates due to the lack of publicly available COVID-19 raw data (confirmed cases and deaths). Values in () for ρb are corrected for the discrepancies in building footprints data due to an incorrect number of buildings. n/a shows lack of publicly available data. (TIF) [file pdig.0000921.s005.TIF]

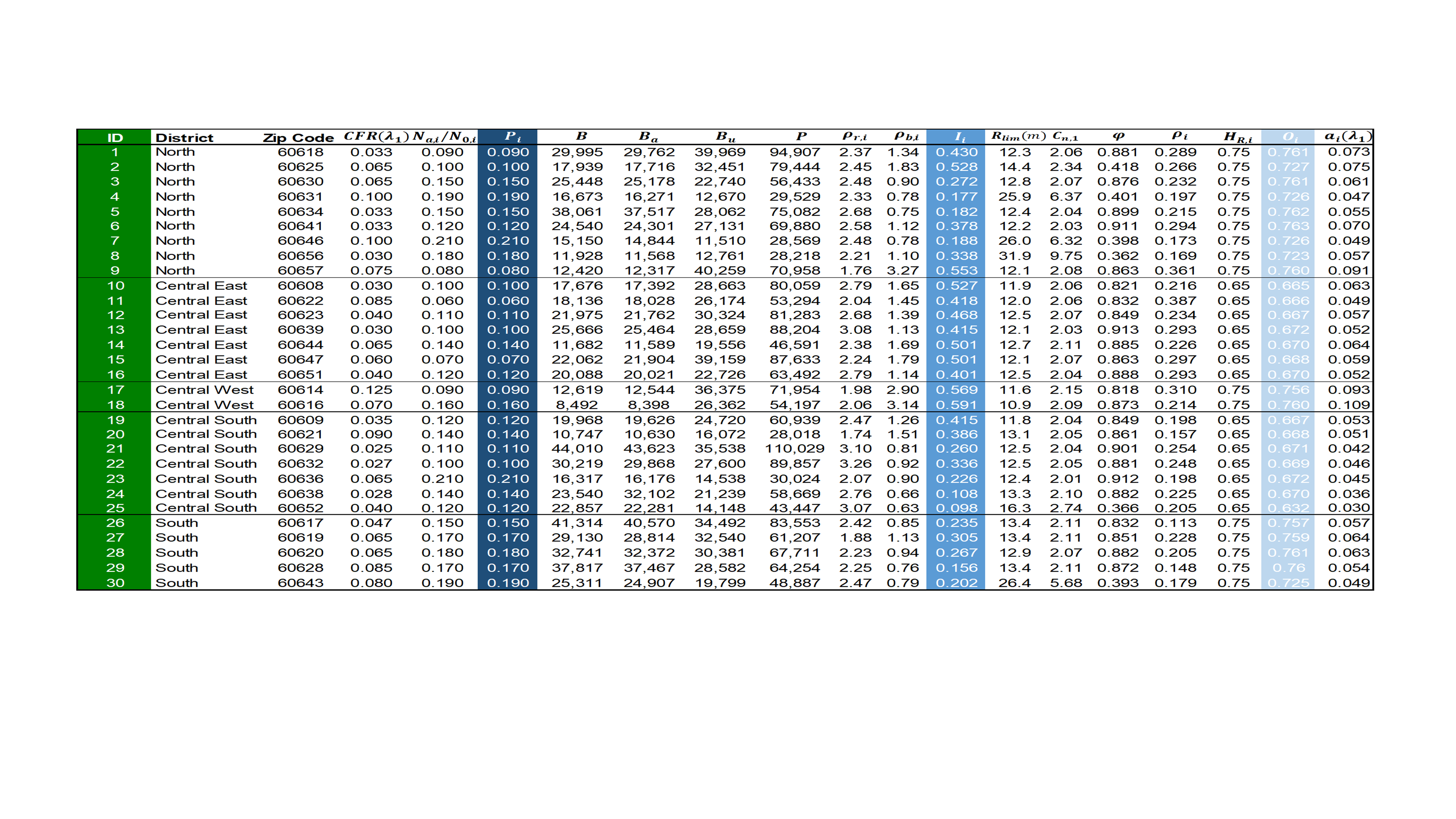

Supplement: S6 Table — (TIF) [file pdig.0000921.s006.TIF]

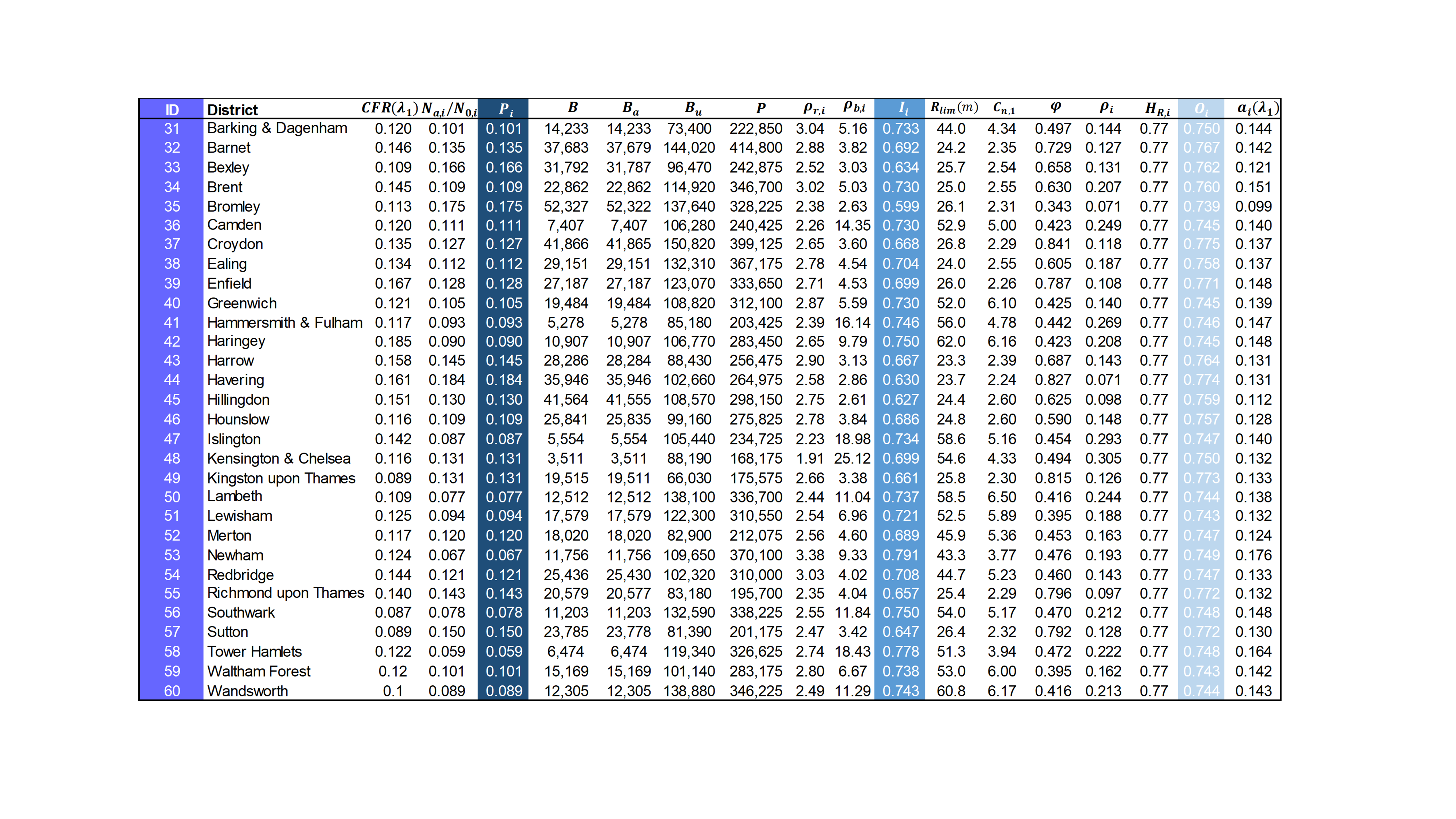

Supplement: S7 Table — (TIF) [file pdig.0000921.s007.TIF]

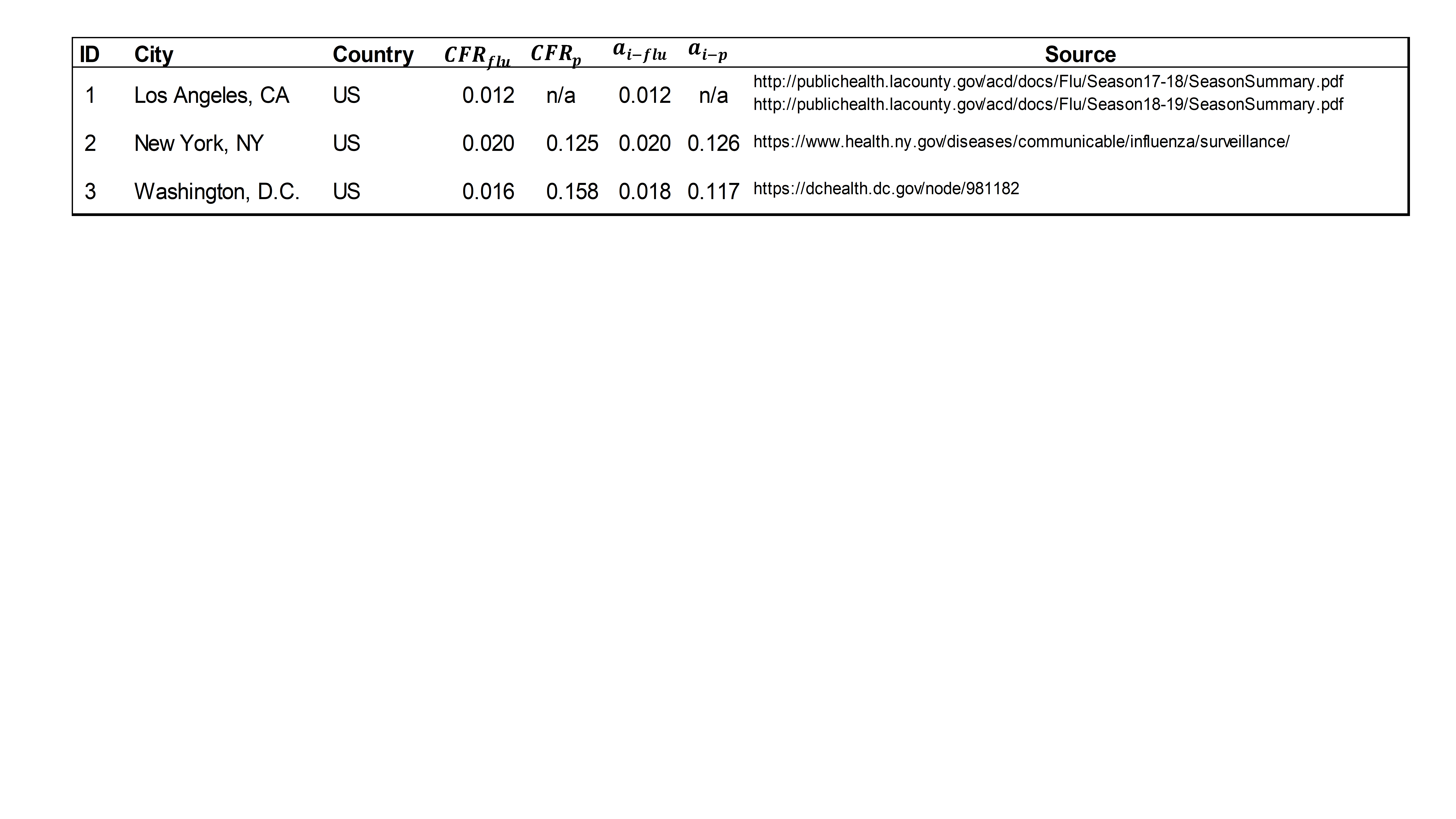

Supplement: S8 Table — (TIF) [file pdig.0000921.s008.TIF]

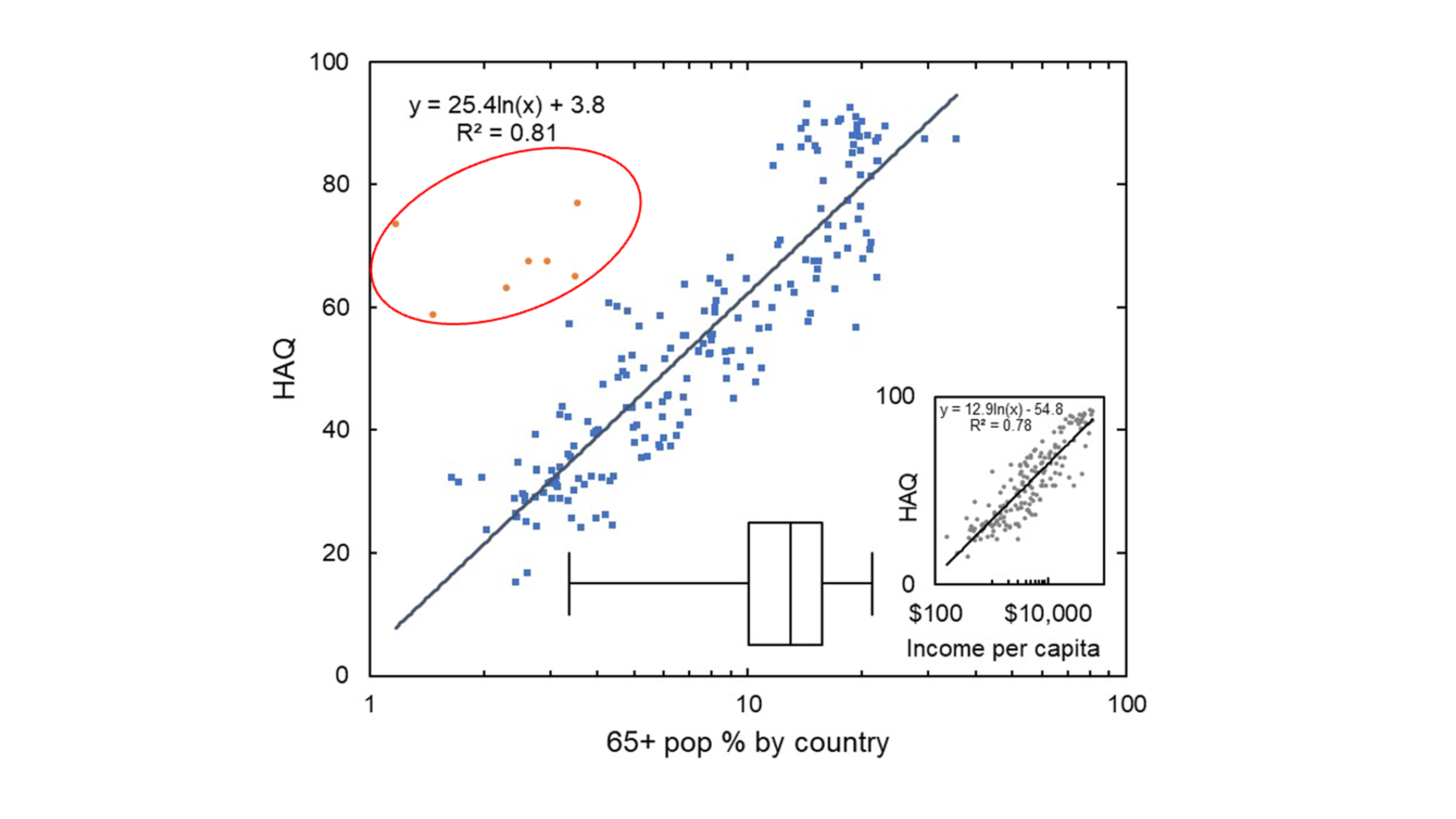

Supplement: S1 Fig — Note that the outliers are Middle East countries with a low < 65yo population but with large investments in their health care system (7). (TIF) [file pdig.0000921.s009.TIF]

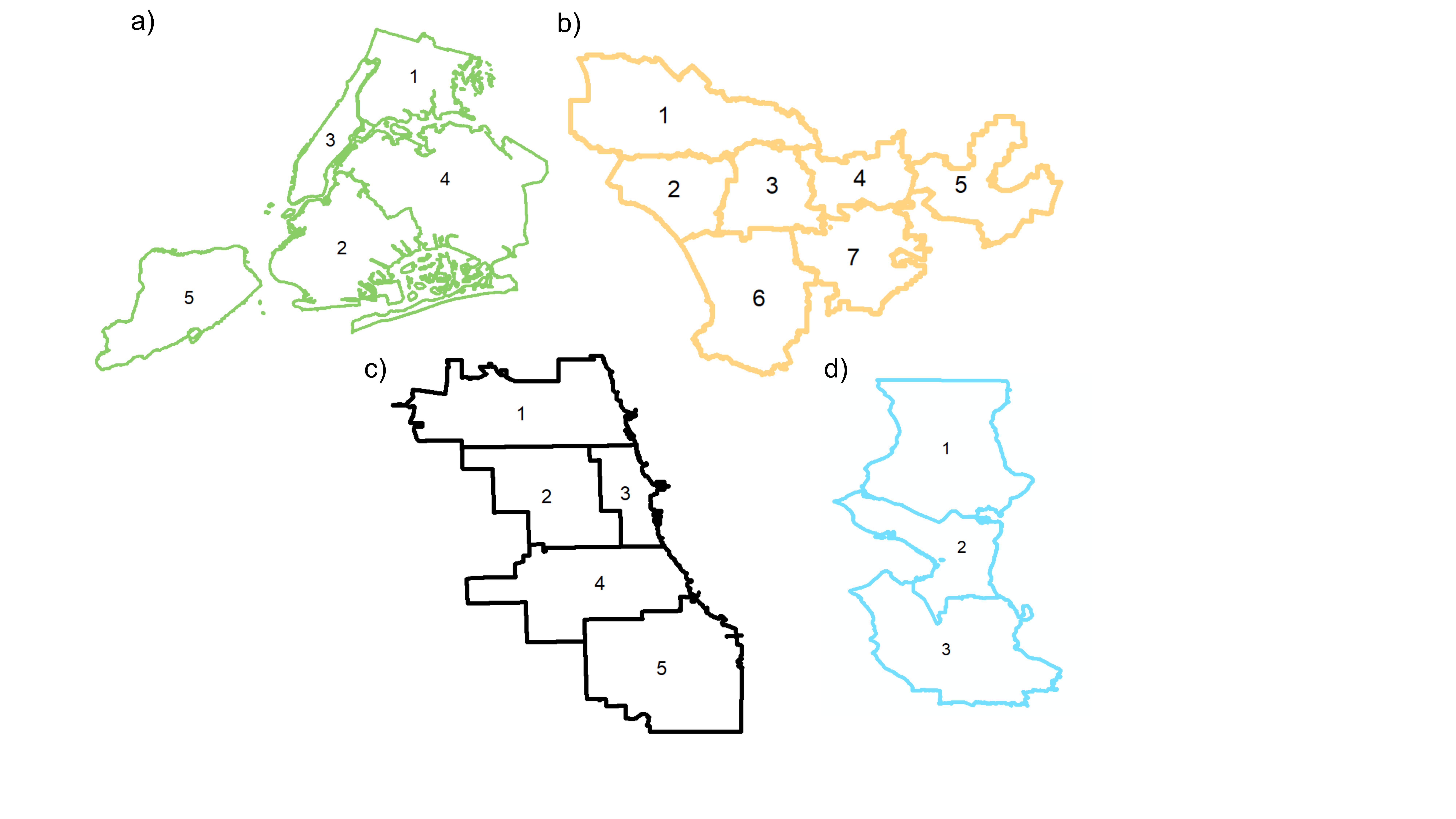

Supplement: S2 Fig — Map created using the Free and Open Source QGIS. Copyright-free countries boundaries data were taken from the world bank (https://datacatalog.worldbank.org/search/dataset/0038272). (TIF) [file pdig.0000921.s010.TIF]

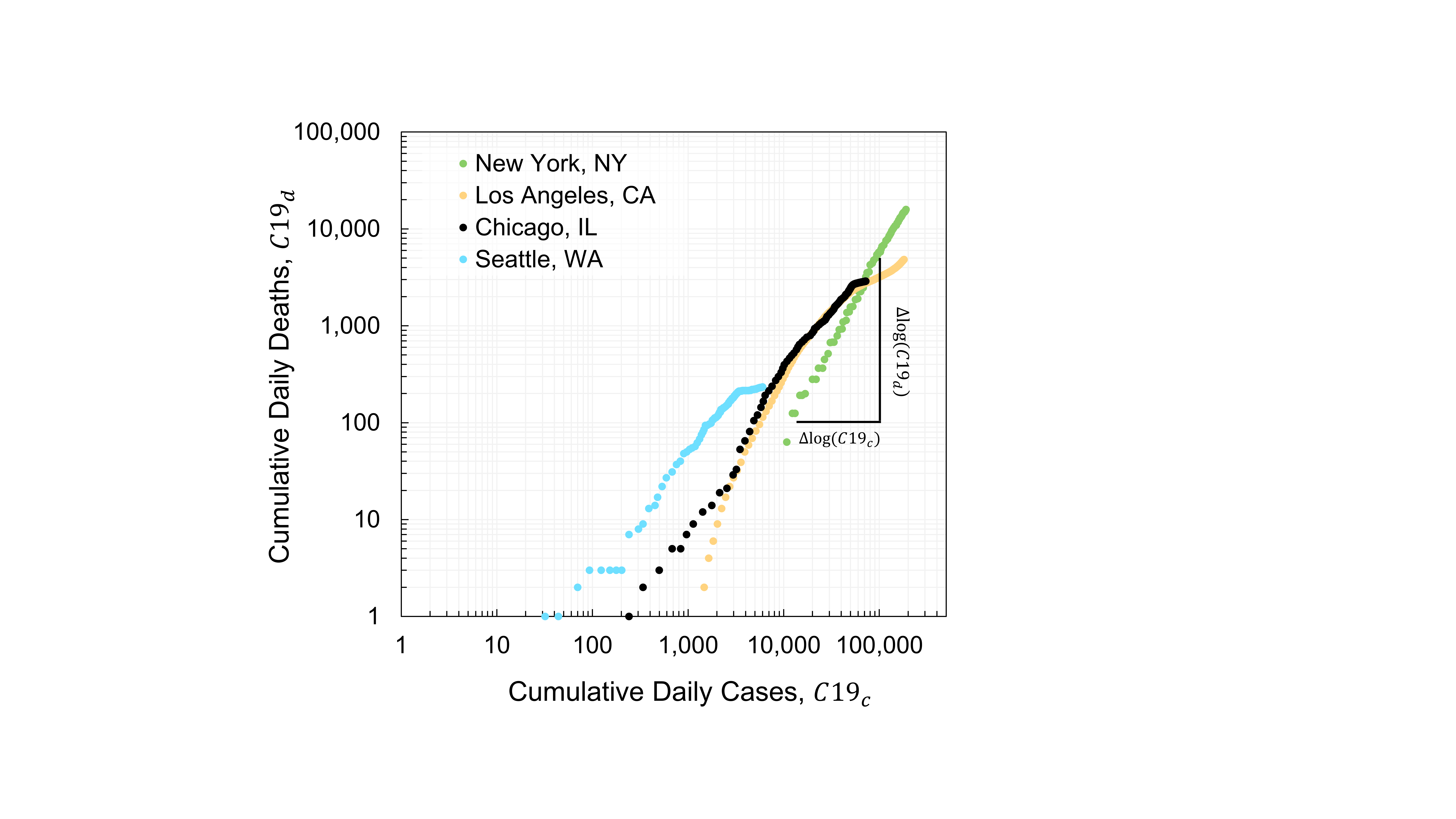

Supplement: S3 Fig — (TIF) [file pdig.0000921.s011.TIF]

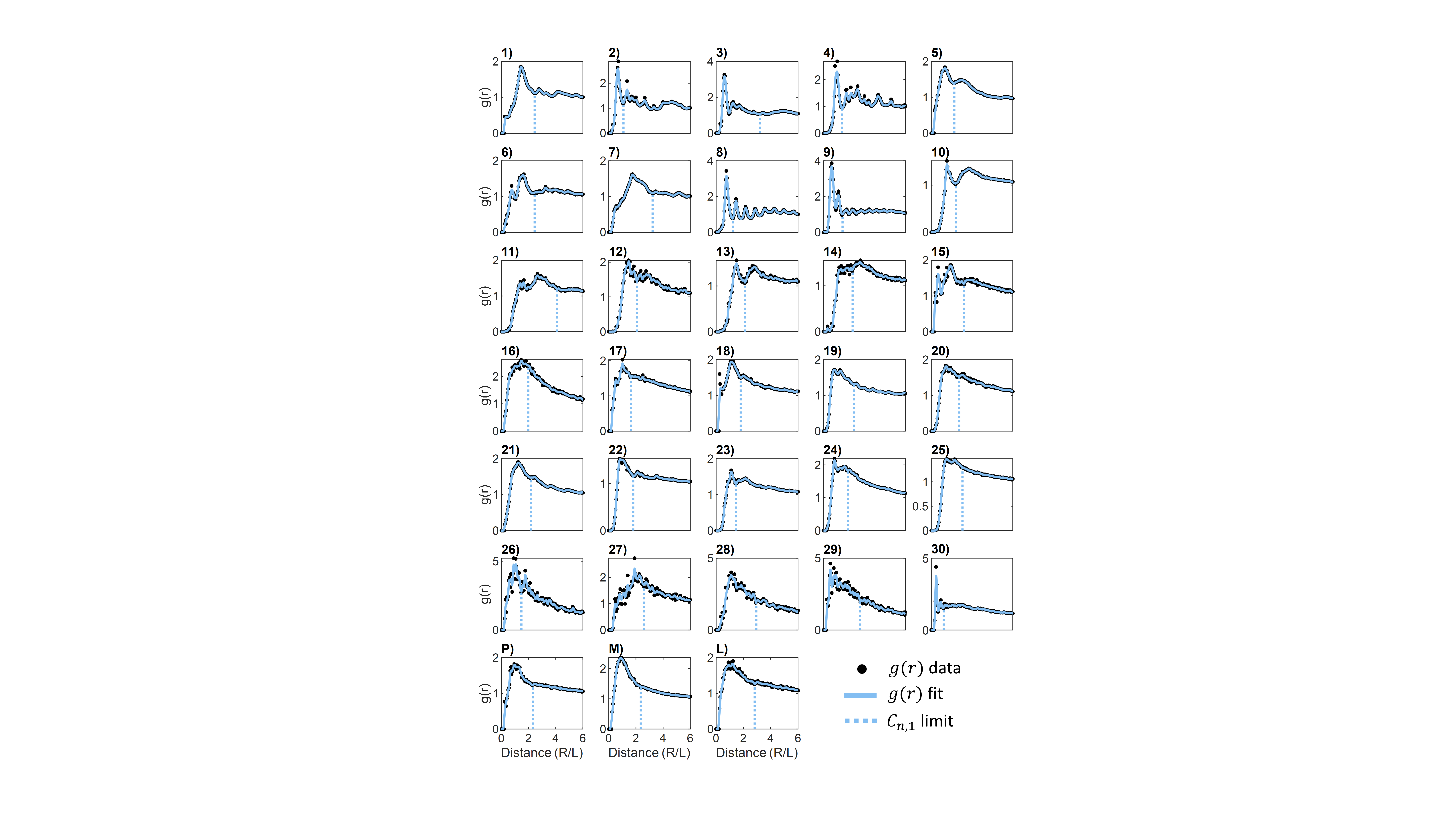

Supplement: S4 Fig — Dashed vertical lines show the distance limits, rmin, used in the determination of order parameter, φ. (TIF) [file pdig.0000921.s012.TIF]

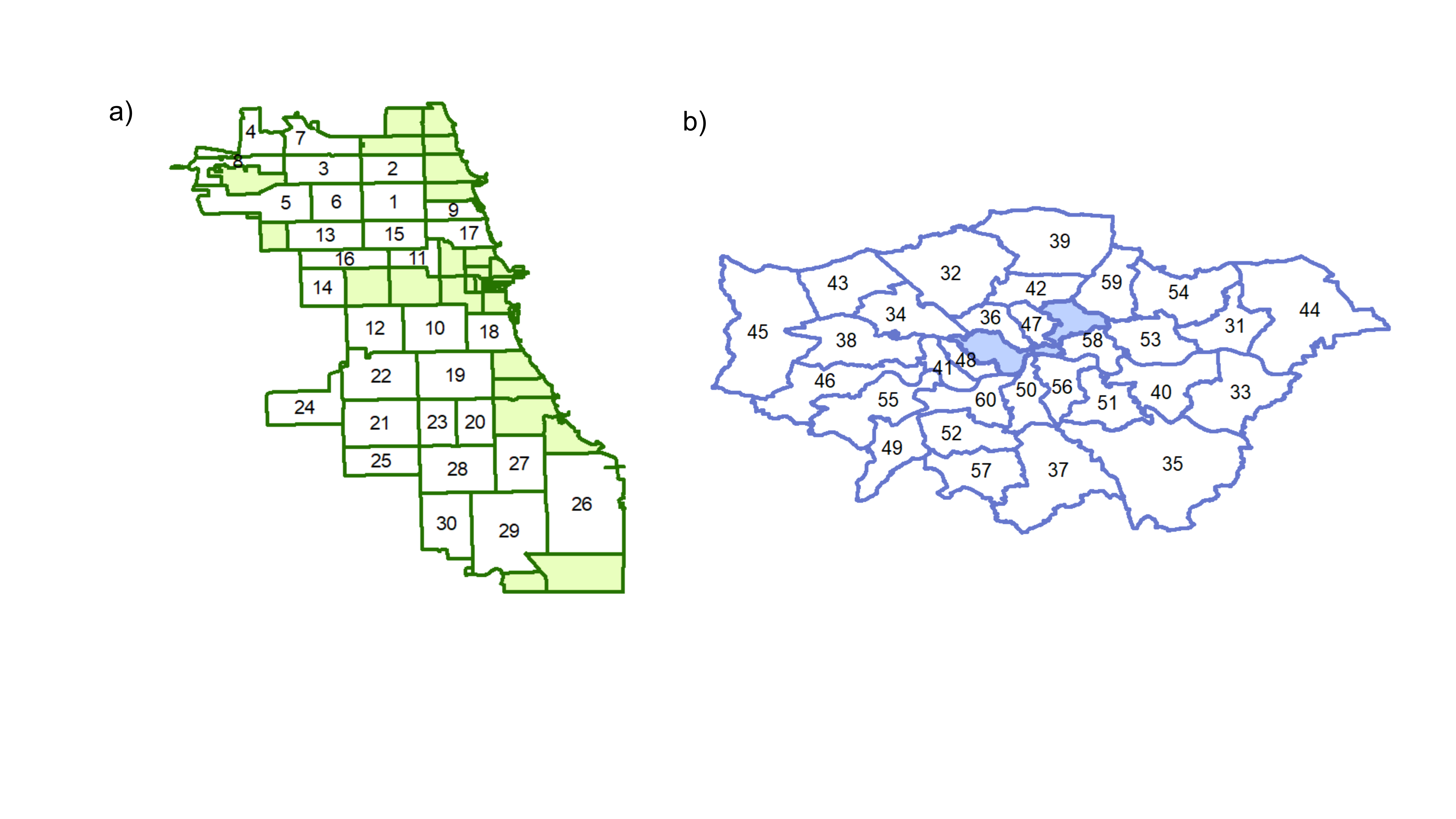

Supplement: S5 Fig — Map created using the Free and Open Source QGIS. Copyright-free countries boundaries data were taken from the world bank (https://datacatalog.worldbank.org/search/dataset/0038272). (TIF) [file pdig.0000921.s013.TIF]

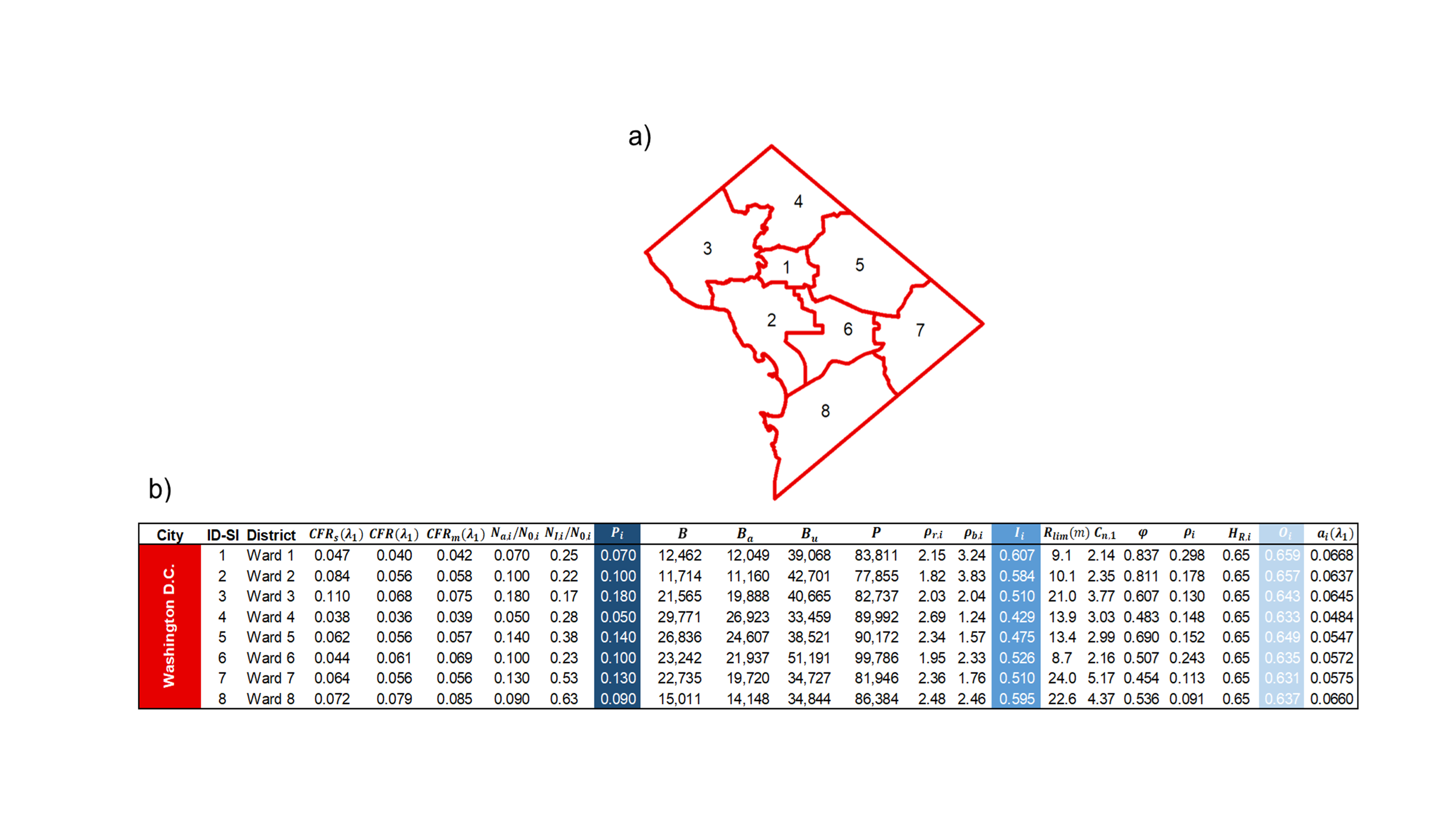

Supplement: S6 Fig — Map created using the Free and Open Source QGIS. Copyright-free countries boundaries data were taken from the world bank (https://datacatalog.worldbank.org/search/dataset/0038272). (TIF) [file pdig.0000921.s014.TIF]

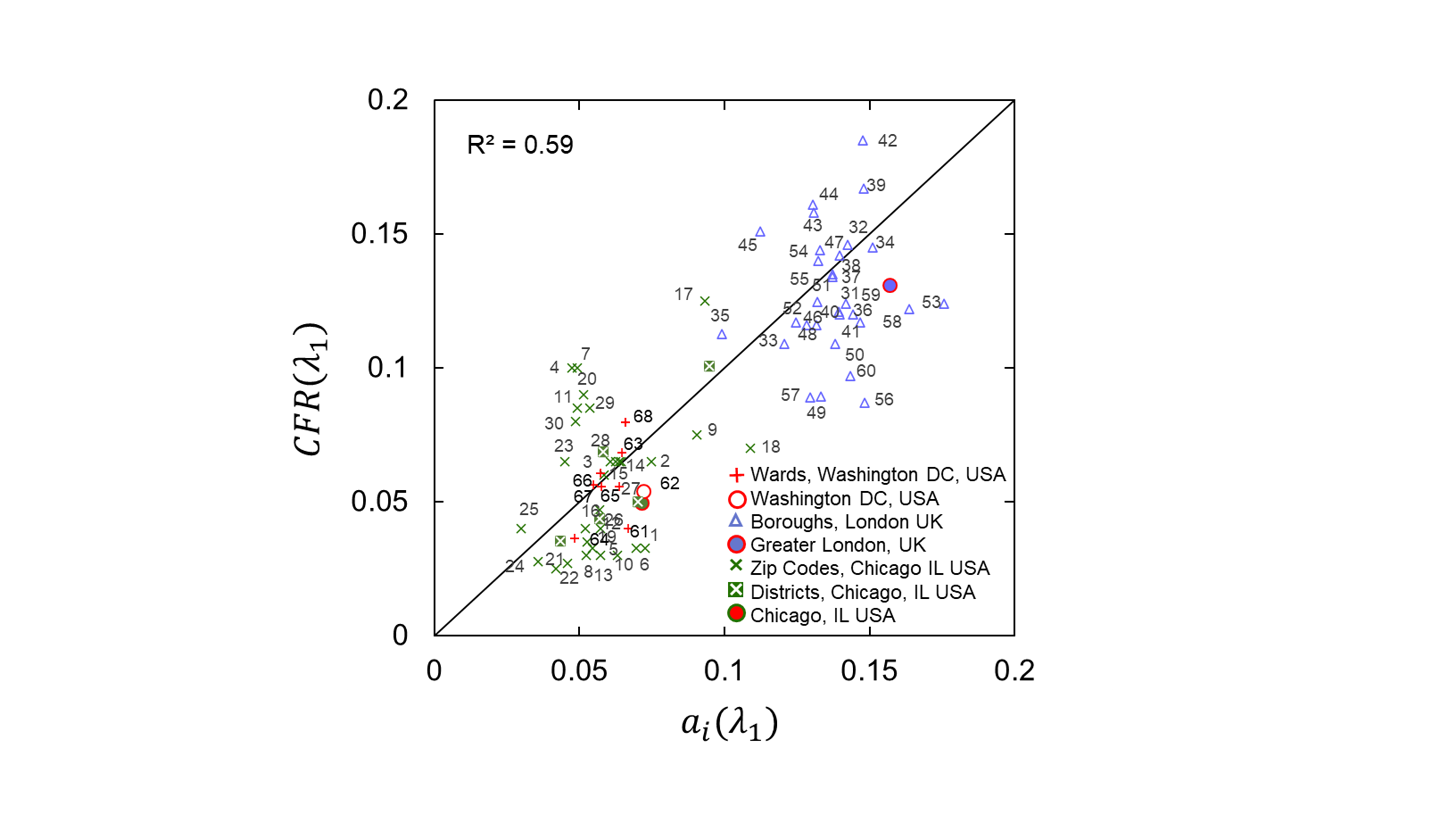

Supplement: S7 Fig — Linear fitting with slope coefficient of unity provides R2=0.59 and RMSE=0.027. For comparison city and city district data are presented in this figure. Predicted CFR values used the urban factor weight parameters from Fig 3, the same as CFR values in Fig 4. (TIF) [file pdig.0000921.s015.TIF]
