# Supplementary material for: Urban environmental and population factors as determinants of COVID-19 severity: A spatially-resolved probabilistic modeling approach
Source: PLOS Digit Health. 2025 Jul 18;4(7):e0000921. doi: 10.1371/journal.pdig.0000921 (PMC12274012; doi:10.1371/journal.pdig.0000921)
Supplement: S1 Text — (DOCX) [file pdig.0000921.s016.docx]

**Supplementary Information**

Urban Environmental and Population Factors as Determinants of COVID-19 Severity: A Spatially-Resolved Probabilistic Modeling Approach

Jacob Roxon^1^, Marie-Sophie Dumont^1,3^, Eric Vilain^1,4^, Mircea T. Sofonea^2^, Roland J.-M. Pellenq^1,5^*

^1^ EpiDaPo Lab - CNRS / George Washington University Children’s National Medical Center, Children’s Research Institute, Washington, District of Columbia, United States of America

^2^ Pathogenesis and Control of Chronic and Emerging Infections (PCCEI, U1058) – University of Montpellier, INSERM, EFS, Univ. Antilles and CHU de Nimes, France

^3^ iGLOBES laboratory, CNRS, ENS-Paris/PSL Université and the University of Arizona – Marshall Building, Tucson, Arizona, United States of America

^4^ School of Medicine, UC-Irvine, Irvine, California, United States of America

^5^ Institut Européen des Membranes, CNRS and University of Montpellier, Montpellier, France

* roland.pellenq@cnrs.fr


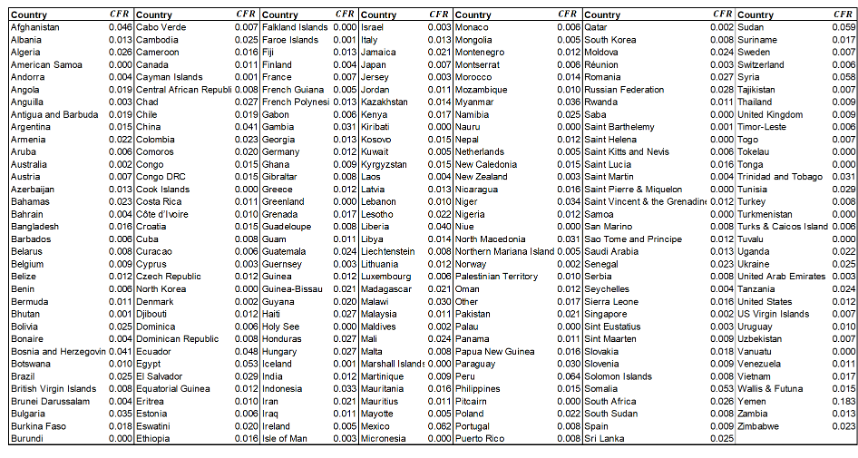


**S1 Table** Cumulative $CFR$ data for countries for period between March 2020 and January 2022 obtained from World Health Organization (WHO).

The objective of this work is to provide a model that provides accurate predictions of the severity of COVID-19—captured with the Case Fatality Ratio $CFR$ (Eq.1, Fig 2a)—by using urban factors derived using Eqs.3-5. Input data for this work is publicly available and has been obtained from healthcare, census, weather and building footprints repositories (S2 Table, S3 Table). Missing or incomplete data for one of the groups meant that we couldn’t use data in our analysis. The only exception were three cities in France, where COVID-19 data was not publicly available, but due to availability of other inputs, we used cities in France to exemplify the impact of the results of our study. For the analysis of data and statistical modeling a standard spreadsheet editing software package provides adequate capabilities to clean and analyze data, which in the context of this study was Microsoft Office Excel. For mapping of data we resorted to using open source QGIS software, but most standard geographical information system (GIS) software package could be used for reproducibility.

$CFR$ is a critical and important measure of the severity of a disease/pathogen which allows to compare apparent lethality across time and space, conditioned upon equal detection rate. As a function of both individual and population-wide risk factors, vulnerabilities and context, it captures a large set of influences from social profiles and habits, including age, health, population density etc. as well as environmental conditions such as temperature, relative humidity, atmospheric pollution, or UV radiation. Therefore, for a given dominant SARS-CoV-2 variant and population (vaccine and/or infection-induced) immunity, the $CFR$ can vary between different geographical locations, including cities or countries (Fig 1, S1 Table). The accurate determination of the $CFR$ has been the topic of many scientific studies and is considered to be a key quantity for targeted emergency and health care infrastructure deployment (1–6). Being a ratio of deaths and confirmed cases of COVID-19, $CFR$ thus is only as accurate as the testing methods and effort to report the number of cases and deaths. At the beginning of the SARS-CoV-2 pandemic spread, there were very limited capabilities to precisely evaluate at the scale of countries the number of infected people with corresponding mortality rates; more importantly, however, these values were still on the rise, which based on the distribution analysis of the COVID-19 data (Figs 2a-b, S3 Fig) appear to have become more stable six to eight months after the beginning of the pandemic (March 2020) signifying the end of the first wave, $\lambda_{1}$. Thus, $\lambda_{1}$ period was selected for the evaluation of $CFR$. Although, other waves of the pandemic should also be studied to understand the underlying factors attributing to variations in $CFR$ for the same geographical location, due to limitations of COVID-19 data (confirmed cases and deaths) at the city level and city district levels, in this study the focus is on the first wave, $\lambda_{1}$ (which for the purposes of this study, depending on the location includes some parts of second wave data between October 2020 and February 2021) and cumulative $CFR$ values from the beginning of the pandemic for the first, second, third, $\lambda_{3}$ (February 2021 – June 2021) and the fourth, $\lambda_{4}$ (July 2021 – January 2022) waves of the COVID-19 pandemic. To offset geographical impact on the definition of length of waves, $\lambda_{i}$, we allowed at least a 4-month term to define each wave of the pandemic. For any COVID-19 data used in the study, the assumption is that all reported data for any given location is for its residents only.


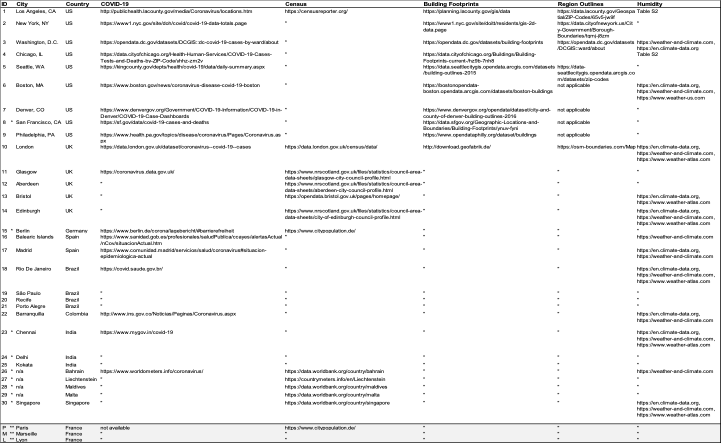


**S2 Table** Sources for data for city districts and cities. Multiple sources for humidity were used to obtain average values presented in S5 Table.


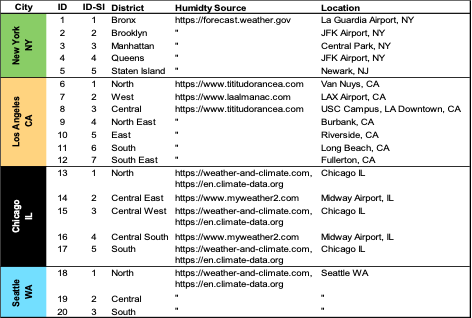


**S3 Table** Sources for humidity data for city districts data used for model optimization (Fig 4a). All sources for each location were used to derive humidity values in S4 Table.


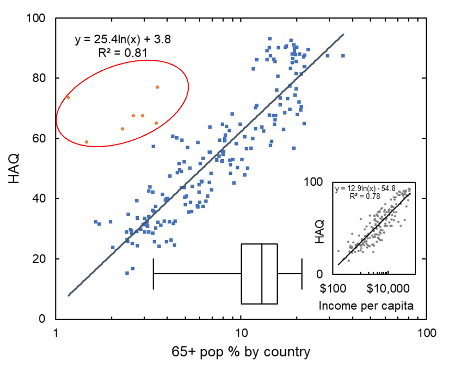


**S1 Fig** Healthcare Access Quality factor at the nation scale for 200+ countries. Note that the outliers are Middle East countries with a low <65yo population but with large investments in their health care system (7)


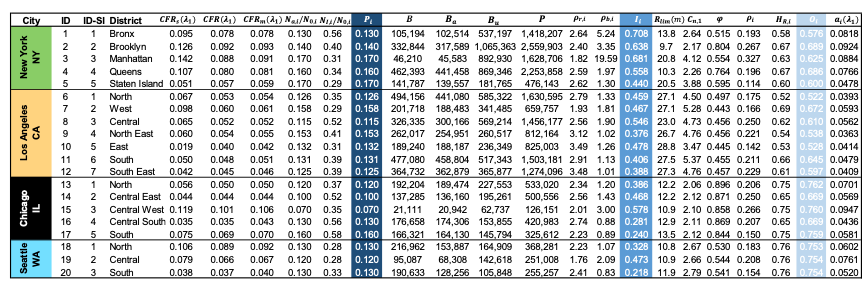


**S4 Table** City Districts measured and predicted $CFR$ data used to derive optimal Urban Factors with their input values. District boundaries are defined using boundaries as shown in S1 Fig.


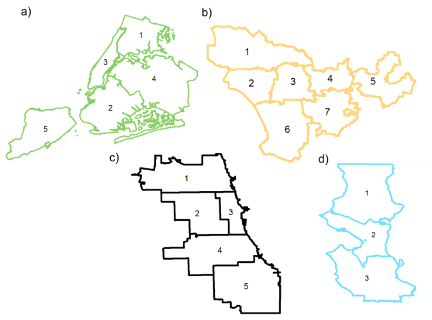


**S2 Fig** (a) New York NY boroughs, (b) Los Angeles CA city and county districts, (c) Chicago IL districts, (d) Seattle WA districts. Map created using the Free and Open Source QGIS. Copyright-free countries boundaries data were taken from the world bank (https://datacatalog.worldbank.org/search/dataset/0038272)


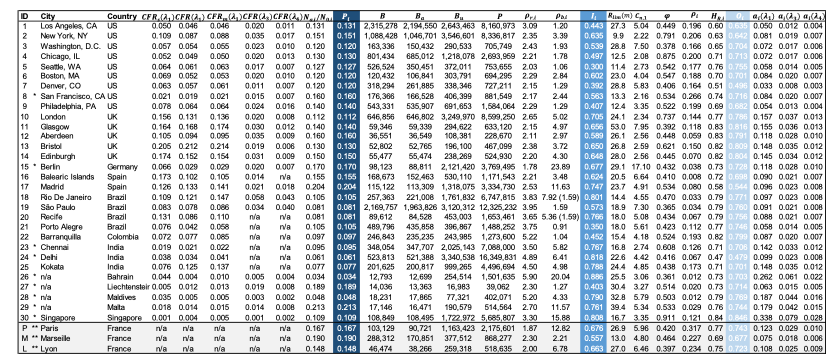


**S5 Table** Cities measured and predicted $CFR$ data with Urban Factors and input values used to derive them. *Outliers in $CFR$ predictions – cities with $CFR$ values close to intrinsic $CFR$, $a_{0}$. **Cities with no $CFR$ estimates due to the lack of publicly available COVID-19 raw data (confirmed cases and deaths). Values in () for $\rho_{b}$ are corrected for the discrepancies in building footprints data due to an incorrect number of buildings. n/a shows lack of publicly available data.


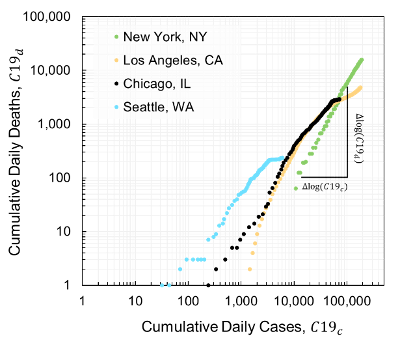


**S3 Fig** $CFR$ distribution capturedvs. the ratio of cumulative daily cases and deaths reported within the first 6-8 months of the COVID-19 pandemic during $\lambda_{1}$ for 5 US cities that were used to evaluate $CFR$ for city districts (S4 Table) demonstrating the exponent in Eq.1 is close to unity.

*Urban Factors.*

The urban factors in this study are categorized into three groups: personal, indoor and outdoor. There are three main sources of input data, which come from weather, census and online maps with building footprints. From the census data (S1 Table), we obtain population size, $P$, population of age 65 or greater, $N_{a}$, population with income of $50,000 or less, $N_{i}$, and average household size $\rho_{r}$, which when combined with the number of buildings allows us to approximate number of housing units ($B_{u}$ per building, $\rho_{b}$ are sufficient to derive Personal, $P_{i}$ and Indoor, $I_{i}$ Factors. To derive Outdoor Factor, $O_{i}$we resort to using annual relative humidity, $H_{r}$ data (S2 Table and S3 Table) for the corresponding to the first wave of COVID-19 period, and building footprints, which are needed to capture 2-D planar building density and quantify city texture with an angular order between local buildings by using a 2-D order parameter, $\varphi$ (8). In the US, building footprints can oftentimes be obtained from city or state’s building and planning departments (S2 Table), but for locations outside the US, we resort to using OpenStreetMap (OSM) repository, since it offers a direct access to building footprints for any mapping layer visible on OSM. To extract city-wide data from OSM that would correspond to COVID-19 data, we leverage OSM boundaries depository (S2 Table). However, sometimes OSM data is not complete leaving empty spaces in areas where one would expect to see multiple parcels of buildings. Thus, when using OSM data, it is important to investigate building footprints data to access its validity. To further clean and prepare data for analysis, we perform building area correction to the number of buildings. Since we are interested in measuring impact of COVID-19 in residential and community areas, we impose a minimum area of 20m² (indicative of non-residential buildings, i.e. garages) for the number of buildings, $B$ used to define corrected number of buildings, $B_{a}$ used in this study. Although, we were able to obtain COVID-19 data for more than 30 cities worldwide, due to building footprints limitations in countries like, India, Argentina, or Columbia, we have not been able to use them. Moreover, some OSM data provides merged buildings instead of individual ones, which underestimates values of $B_{a}$ and overestimates of $\rho_{b}$ used in determining indoor factors for Rio De Janeiro and Recife cities in Brazil, where at the city scale, one would expect to see lower values for $\rho_{b}$. To offset the error, we adopt average $\rho_{b}$ values from São Paulo, Brazil, which upon visual mapping verification, we would expect to have, on average, similar range to other major cities in Brazil.

The striking resemblance in texture between urban environments and molecular structure of polycrystalline material at an atomic scale can be established with the help of appropriate visualization techniques. To extract statistical characteristics of short and long-range city texture, we employ radial distribution function, also known as pair (or two-points) correlation function. Denoted by $g\left( r \right)$, it provides an isotropic homogenous picture of an anisotropic inhomogeneous medium by averaging the local density over time and space domains. In the context of cities and buildings, it can be thought of as a mechanism of describing density variation at a given distance from the reference building. As soon as local density deviates from the average density of a system, peaks in the distribution eventuate; in statistical particle physics terms applied to cities, this is explained as the probability of finding a building at distance $r$ from the reference building relative to randomly distributed system of buildings that at long distance converges to unity, i.e being normalized by the averaged total density of buildings within the circle defined by maximum radius, $r_{max}$. which is the limiting radius for $g\left( r \right)$ analysis—distance at which the function convergences to unity—here defined to be $15L$ where $L$ is the average building size for a city calculated using the following equation:

$L=exp\left( \frac{1}{2N}\sum_{i=1}^{N} log\left( A_{i} \right) \right)$ (Eq. S1)

where $N$ is the total number of buildings in the city and $A_{i}$ is the area of building $i$. The buildings size distributes follow a lognormal distribution. However, for many cities outside the US, it shows a significant tail for large values of L, which is representative of the fact that in older cities there are more buildings with larger areas – indicative of terraced housing. The sum of buildings projected 2-D areas divided by the city total surface gives the city’s build-up ratio, average density of buildings, $\rho$. In order to quantify more accurately local average density of buildings, we utilize an average, $\rho_{city}$, from the distribution of density values as defined by:

$\rho_{city}=exp\left( \frac{1}{N}\sum_{i=1}^{N} log\left( \frac{C_{n+1}^{r_{max}}}{\pi r_{max}^{2}} \right) \right)$ (Eq. S2)

where $C_{n+1}^{r_{max}}$ is the total number of buildings in circular area of radius $r_{max}$. With such defined average density, $g\left( r \right)$ captures the local deviation from it in the following form:

$g\left( r \right)=\frac{1}{N}\sum_{i=1}^{N} \frac{n_{i}\left( r+dr \right)-n_{i}\left( r \right)}{\rho_{city}2\pi r\times dr}$ (Eq. S3)

where $n_{i}\left( r \right)$ denotes the number of buildings within the radial distance $r$ from building $i$, and $dr$ is distance increment, which for $g\left( r \right)$ calculations we chose to be 5% of the average building size, $L$. We find that despite similar geographical location, distribution of buildings in a given ward can exhibit different characteristics of local order than that in other wards of the same city. To further quantify local texture patterns on the path of exploring meaningful ways of characterizing city texture, we enrich our city characterization toolbox with coordination number and order parameter.

In the context of buildings, coordination number, $C_{n}$ defines the number of neighboring buildings that are within a specific distance, $r_{min}$, from the building of reference. Although, these can be counted individually and then averaged, $C_{n}$ can also be obtain directly from the $g\left( r \right)$ function, using its integral in the form:

$C_{n}=2\pi\rho_{city}\int_{0}^{r_{min}} g\left( r \right)dr$ (Eq. S4)

The approach for selecting $C_{n}$ should be evaluated based on its application, which depending on desired accuracy may lead to different results. Here, the application is defining average local configuration of buildings as captured by $g\left( r \right)$ thus leading to our use of Eq. S4, where $r_{min}$ is the first local minimum in the $g\left( r \right)$ distribution following the main peak (i.e. one with the maximum $g\left( r \right)$ value). Due to variability in local city texture between zip codes or wards within a given city, integral of the first peak may not always lead to $C_{n}>$ 1, which is required to be able to obtain angular order parameter with a minimum configuration of 3 buildings, or 2 neighbors. If such situation exists, we proceed with the next local minimum in the distribution of $g\left( r \right)$ until $C_{n}>$ 1 has been obtained. With such defined $C_{n}$ we proceed with calculations of the second city texture value, order parameter. Exact $r_{min}$ values used to define the first peak are presented in S4-S7 Tables, with $g\left( r \right)$ functions visualized for cities and their first peaks for calculations of order parameters in S4 Fig.

Order parameter, $\varphi$, which is a general Mermin 2-D order parameter, designed in condensed matter physics to quantify the deviation from symmetrical order of two-dimensional crystals as:

$\varphi=1/{N_{a}}\left| \sum_{k=1}^{N_{a}} exp\left( \mathfrak{I}\vartheta_{k} \right) \right|$ (Eq. S5)

where $m$ is the number of atoms in the first shell, $N_{a}$, the number of independent angles $\vartheta_{k}$ between the atom and its neighbors. For a perfectly ordered (symmetrical) system, $\varphi=1$ for a system with a perfect m-fold orientational ordering. Applied at the city scale, $\varphi$ characterizes the average angular distortion of buildings compared to a perfect angular local order of a city at fixed $m=C_{n}$ within the first shell distance determined from the integral of $g\left( r \right)$ (Eq. S4):

$\varphi=\frac{1}{N}\sum_{j=1}^{N} \frac{1}{N_{a}\left( j \right)}\left| \left( \sum_{k=1}^{N_{a}\left( j \right)} exp\left( iC_{n}\vartheta_{k} \right) \right) \right|$ (Eq. S6)

where, $N$ is the number of buildings. So defined, $\varphi$ = 1 represents a city in which all buildings at short distance have the same number of neighbors exhibiting angular periodicity, $2\pi/{C_{n}}$; whereas any deviation from unity in this short-range city order parameter is representative of both local angular distortions of neighboring buildings, and local variations of number of neighbors that affects the number of independent angles $N_{a}\left( j \right)$ for each building $j=1,N$. At the scale of cities, $\varphi$ becomes the arithmetic mean of order parameters values for all buildings. While it has been previously established that much like molecular structures cities exhibit a distinct long-range texture, which varies from gas- and liquid/glass- to crystal- like (8), here we also find that despite similar geographical location, distribution of buildings in different parts of the city (i.e. wards or districts) can exhibit different characteristics of local order (i.e. Brooklyn and Bronx in New York City, NY).

Although, gas-like (European) cities lack the expected $g\left( r \right)$ characteristics used to identify the first minimum or simply have no minima, here we utilize the distance, $r_{peak}^{g\left( r \right)}$ at which $g\left( r \right)$ reaches its first peak – a characteristic distance between one building and its nearest neighbors. This distance correlates with $r_{min}^{g\left( r \right)}$ for cities where it is possible to identify it and can be modeled using a linear correlation (9):

$r_{min}^{g\left( r \right)}={1.35\times r}_{peak}^{g\left( r \right)}$ (Eq. S7)

Such linear correlation can be explained by the characteristic street width, which on average limits the local buildings to the nearest 2 neighbors. With such approach, we can identify $r_{min}^{g\left( r \right)}$ for any city or zip code, subsequently allowing us to derive coordination number for the first shell of neighbors. This is an important step, because distance $r_{min}^{g\left( r \right)}$ is a critical input for quantifying $\varphi$, which is used as one of the outdoor urban factor parameters.

In the present work, we seek at identifying differences in the COVID-19 spread in a given city captured through $CFR$. We therefore derive $g\left( r \right)$ functions for every location used in this work to obtain $\varphi$s, among other variable used in the determination of Urban Factors (S4 Table - S7 Table). However, for the calibration of weight parameters in equations 2-4 only city district ($N=20$) from S3 Table have been used. This allowed sufficient sample of uncorrelated data from various geographical regions to be used for validation of the model ($N=98$ for cities, zip codes and boroughs). The model parameters have been optimized by minimizing the error between predicted and measured $CFR\left( \lambda_{1} \right)$ values using standard statical methods. Although, it is common to apply sum of square errors when quantifying errors in regression analyses, here we adopted a sum of absolute errors, $\varepsilon$ in the form of:

$\varepsilon=\sum_{i=1}^{n=20} \left| CFR_{i}\left( \lambda_{1} \right)-a_{i}\left( \lambda_{1} \right) \right|$ (Eq. S8)

where $i$ is the city district ID from S4 Table. Since $CFR$ values are fractions, differences between any predicted and measured values lead to small fractions and thus squaring the difference would lead to even smaller values thereby introducing bias to any optimization approach that is trying to minimize the sum of errors. As such the absolute sum $\varepsilon$ provides a more stable optimization parameter. We minimize $\varepsilon$ using a non-linear generalized reduced gradient (GRD) method with constraint convergence of 0.00001, forward derivates population size of 1000. With such defined parameters, we obtain optimized urban factors and intrinsic $CFR$, $a_{0}\left( \lambda_{1} \right)$ (Fig 3).


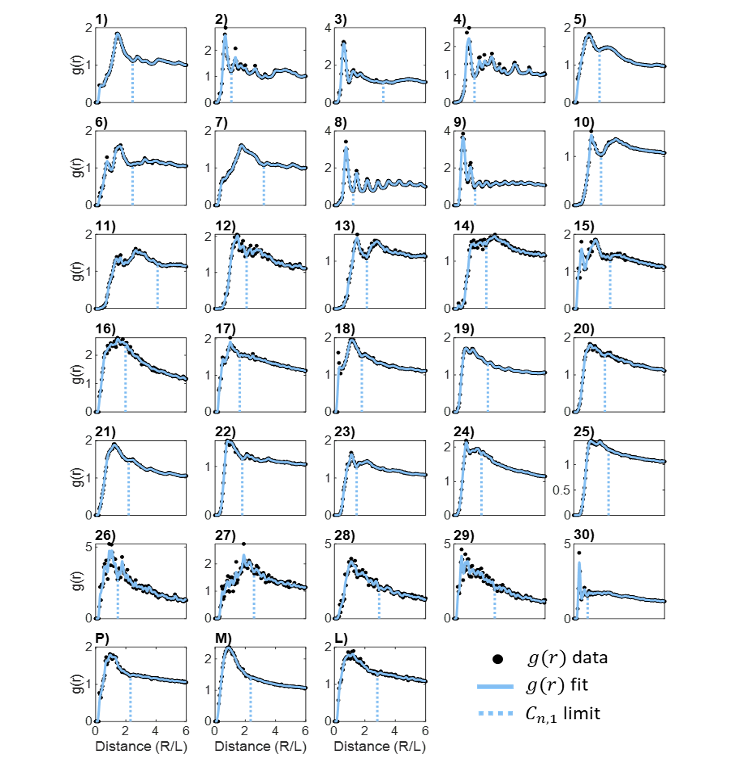


**S4 Fig** Radial distribution function, $g\left( r \right)$, for cities in S5 Table. Dashed vertical lines show the distance limits, $r_{min}$, used in the determination of order parameter, $\varphi$.


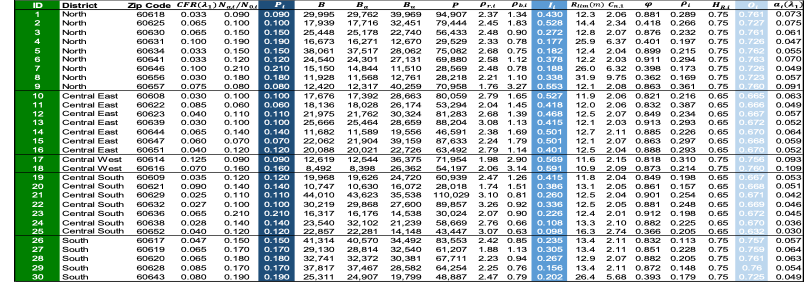


**S6 Table** Chicago IL, USA measured and predicted $CFR$ data with Urban Factors and input values used to derive them at the zip code level (S5a Fig), which were merged to form Chicago districts (S2d Fig)


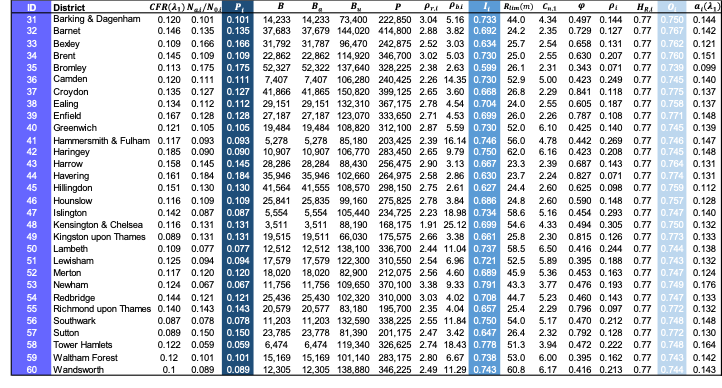


**S7 Table** London UK measured and predicted $CFR$ data with Urban Factors and input values used to derive them at the level of boroughs (S5b Fig).


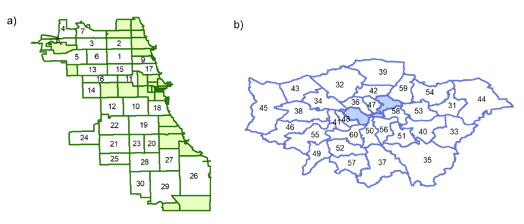


**S5 Fig** (a) Chicago IL, USA zip codes and (b) Greater London UK boroughs. Map created using the Free and Open Source QGIS. Copyright-free countries boundaries data were taken from the world bank (https://datacatalog.worldbank.org/search/dataset/0038272)

To verify the validity of the model and its multi-spatial scale global application, we test the correlation between predicted and measured population adjusted $CFR_{pop}$ values for 118 location worldwide (Fig 4b). Although, we find that $CFR_{pop}\left( \lambda_{1} \right)$ for several cities cannot be predicted using the model, we identify those as places either isolated territories (island or small countries) or regions that were quick to adopt social distancing and any lockdown measures during the first wave, $\lambda_{1}$, of the COVID-19 pandemic. It is worth noting that any country used in this study for model verification as listed in S5 Table, due to its small population size and area, is treated as a city and thus compared to any other city listed in the table. To further study verify the multi scale application, we introduce data for 30 zip codes from Chicago IL, USA (S7a Fig) and 30 boroughs from Greater London region in the UK (S7b Fig) as well as 8 wards from Washington D.C. USA (S6 Fig), which due to their low population values ($100,000$) were not used in the optimization of the model in Eq. 2. Not only this data presents that measured $CFR_{pop}$) varies significantly across different locations, but it also shows that the Urban Factors model can predict accurately $CFR_{pop}$ values at the scale smaller than city and district levels.


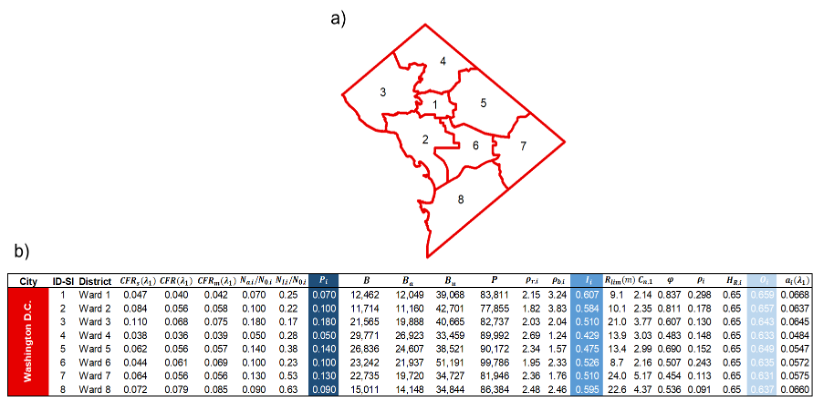


**S6 Fig** Washington D.C. USA wards data showing a) geographical boundaries of wards and b) measured and predicted $CFR$ with their input values. Map created using the Free and Open Source QGIS. Copyright-free countries boundaries data were taken from the world bank (https://datacatalog.worldbank.org/search/dataset/0038272)


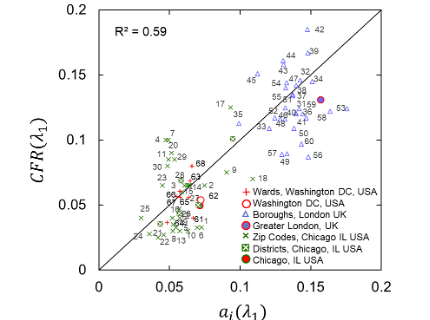


**S7 Fig** Urban Factors model predications showing a comparison between measured and predicted $CFR\left( \lambda_{1} \right)$ from Eq.5 for 30 zip codes in Chicago IL, USA (S6 Table) and 30 boroughs in London, UK (S7 Table), 8 wards in Washington D.C. USA between March 2020 and September 2020. Linear fitting with slope coefficient of unity provides $R^{2}=0.59$ and $RMSE=0.027$. For comparison city and city district data are presented in this figure. Predicted $CFR$ values used the urban factor weight parameters from Fig 3, the same as $CFR$ values in Fig 4.


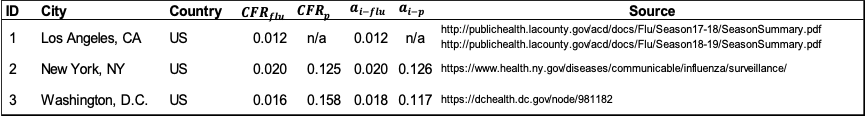


**S8 Table.** Measured and predicted $CFR$ data for pneumonia and flu with sources for each location data needed to derive the values.

**References**

1. Hill AL. The math behind epidemics. Phys Today. 2020 Nov 1;73(11):28–34.

2. Fan G, Yang Z, Lin Q, Zhao S, Yang L, He D. Decreased Case Fatality Rate of COVID-19 in the Second Wave: A study in 53 countries or regions. Transbound Emerg Dis. 2021 Mar;68(2):213–5.

3. Chang CS, Yeh YT, Chien TW, Lin JCJ, Cheng BW, Kuo SC. The computation of case fatality rate for novel coronavirus (COVID-19) based on Bayes theorem. Medicine (Baltimore). 2020 May 22;99(21):e19925.

4. Gupta S, Kumar Patel K, Sivaraman S, Mangal A. Global Epidemiology of First 90 Days into COVID-19 Pandemic: Disease Incidence, Prevalence, Case Fatality Rate and Their Association with Population Density, Urbanisation and Elderly Population. J Health Manag. 2020 Jun 1;22(2):117–28.

5. Narayanan CS. A novel cohort analysis approach to determining the case fatality rate of COVID-19 and other infectious diseases. PLOS ONE. 2020 Jun 15;15(6):e0233146.

6. Neil M, Fenton N, Osman M, McLachlan S. Bayesian network analysis of Covid-19 data reveals higher infection prevalence rates and lower fatality rates than widely reported. J Risk Res. 2020 Aug 2;23(7–8):866–79.

7. Haakenstad A, Yearwood JA, Fullman N, Bintz C, Bienhoff K, Weaver MR, et al. Assessing performance of the Healthcare Access and Quality Index, overall and by select age groups, for 204 countries and territories, 1990–2019: a systematic analysis from the Global Burden of Disease Study 2019. Lancet Glob Health. 2022 Dec 1;10(12):e1715–43.

8. Sobstyl JM, Emig T, Qomi MJA, Ulm FJ, Pellenq RJM. Role of City Texture in Urban Heat Islands at Nighttime. Phys Rev Lett. 2018 Mar 9;120(10):108701.

9. Manav IB, Roxon J, Ulm FJ, Gregory J, Kirchain R. Texture-Informed Approach for Hurricane Loss Estimation: How Discounting Neighborhood Texture Leads to Undervaluing Wind Mitigation. Nat Hazards Rev. 2022 Nov 1;23(4):05022006.

| Glossary | |
| --- | --- |
| Term | Definition |
| $\boldsymbol{CFR}$ | Case Fatality Ratio, $CFR=d/c$ |
| $\boldsymbol{CF}\boldsymbol{R}_{\boldsymbol{pop}}$ | Adjusted for population $CFR$ |
| $\boldsymbol{CF}\boldsymbol{R}_{\boldsymbol{flu}}$ | Case Fatality Ratio for flu |
| $\boldsymbol{CF}\boldsymbol{R}_{\boldsymbol{p}}$ | Case Fatality Ratio for pneumonia |
| $\boldsymbol{a}$ | Algebraic proportionality constant of $CFR$ |
| $\boldsymbol{a}_{\boldsymbol{0}}$ | Intrinsic $CFR$ |
| $\boldsymbol{\lambda}_{\boldsymbol{\#}}$ | Covid-19 pandemic wave number #, i.e. first wave = $\lambda_{1}$ |
| $\boldsymbol{k}$ | Exponent factor of $CFR$ |
| $\boldsymbol{C}\boldsymbol{19}_{\boldsymbol{c}}$ | Covid-19 infected people, $C{19}_{c}=c$ |
| $\boldsymbol{C}\boldsymbol{19}_{\boldsymbol{d}}$ | Covid-19 deaths, , $C{19}_{d}=d$ |
| $\boldsymbol{U}_{\boldsymbol{i}}$ | Model Urban Factor |
| $\boldsymbol{w}_{\boldsymbol{i}}$ | Model Weight Parameter |
| $\boldsymbol{P}_{\boldsymbol{i}}$ | Model Urban Personal Factor |
| $\boldsymbol{w}_{\boldsymbol{A}}$ | Weight factor for inhabitants of age, $\left( A,i \right)\geq65$ years old |
| $\boldsymbol{w}_{\boldsymbol{I}}$ | Weight factor for an annual income$,i\leq\$50,000$ |
| $\boldsymbol{I}_{\boldsymbol{i}}$ | Model Urban Indoor Factor |
| $\boldsymbol{w}_{\boldsymbol{\rho}_{\boldsymbol{b}}}$ | Weight factor for building density, $\rho_{b,i}$ (number of housing units per building) |
| $\boldsymbol{w}_{\boldsymbol{\rho}_{\boldsymbol{r}}}$ | Weight factor for resident density $\rho_{r,i}$ (household size, which is the number of residents per housing unit) in region $i$ |
| $\boldsymbol{O}_{\boldsymbol{i}}$ | Model Urban Outdoor Factor |
| $\boldsymbol{w}_{\boldsymbol{\rho}}$ | Weight factor for the planar density of building footprints, $\rho_{i}$ (ratio between total area of buildings and area of region $i$) |
| $\boldsymbol{g}\left( \boldsymbol{r} \right)$ | 2-D building-building pair correlation function |
| $\boldsymbol{r}_{\boldsymbol{min}}$ | First local minimum distance in the $g\left( r \right)$ distribution following the main peak (i.e. one with the maximum $g\left( r \right)$ value), $r_{min}=\boldsymbol{R}_{\boldsymbol{lim}}$ |
| $\boldsymbol{L}$ | Average building size for a city |
| $\boldsymbol{\rho}$ | Average density of buildings, i.e. city’s build-up ratio |
| $\boldsymbol{C}_{\boldsymbol{n}}$ | Coordination number of Buildings, i.e. local number of buildings, $C_{n}=\boldsymbol{C}_{\boldsymbol{n,1}}$ |
| $\boldsymbol{\varphi}$ | Order Parameter of Buildings |
| $\boldsymbol{w}_{\boldsymbol{\varphi}}$ | Weight factor for the order parameter of buildings, $\varphi_{i}$ |
| $\boldsymbol{H}_{\boldsymbol{R}}$ | Relative Humidity |
| $\boldsymbol{w}_{\boldsymbol{H}_{\boldsymbol{R}}}$ | Weight factor for the relative humidity which is assumed constant at the city-scale |
| $\boldsymbol{B}$ | Number of buildings |
| $\boldsymbol{B}_{\boldsymbol{a}}$ | Corrected number of buildings (excluded buildings below 20m² indicative of non-residential buildings, i.e. garages, sheds) |
| $\boldsymbol{P}$ | Population size |
| $\boldsymbol{N}_{\boldsymbol{a}}$ | Population of age 65 or greater |
| $\boldsymbol{N}_{\boldsymbol{i}}$ | Population with income of $50,000 or less |
| $\boldsymbol{\rho}_{\boldsymbol{r}}$ | Average household size |
| $\boldsymbol{B}_{\boldsymbol{u}}$ | Number of housing units per building |
