# Supplementary material for: Urban environmental and population factors as determinants of COVID-19 severity: A spatially-resolved probabilistic modeling approach
Source: PLOS Digit Health. 2025 Jul 18;4(7):e0000921. doi: 10.1371/journal.pdig.0000921 (PMC12274012; doi:10.1371/journal.pdig.0000921)
Supplement: Attachment — Submitted filename: Response-to-reviewers_covidpaper_April_2024-final.pdf [file pdig.0000921.s019.pdf]

Dr. Roland J.-M. Pellenq
Director of Research with CNRS (DRCEx)
Email: roland.pellenq@cnrs.fr
Tel: (+33) 6 62 92 28 33

Response to Reviewers

Montpellier, April 5th 2024

We would like to thanks the 3 reviewers for giving us the opportunity to improve our manuscript through their genuine, sound comments and remarks.

Dr. Roland Pellenq

Reviewer #1: The paper has the potential for citation but needs improvements.

1/ The title should be changed to "Impact of urban environment and population characteristics on COVID-19 severity."
□ We do thank the reviewer for this suggestion and understand how a more general statement may be seen as more adequate for an article, however, after careful consideration of the manuscript, we believe that introducing the scope of this work in the form of a question "How do Urban Environment and Population Characteristics Control the Severity of COVID-19?" as the title, allows us to access more effectively the audience of this journal and address the answer "Toward Spatially-Resolved Epidemiology" that reflects the core of this work as a methodology that can be extended to various airborne infections at a global level.

2/ The introduction should be improved to better explain the work's motivation and methodology characteristics. The research gap in the introduction should be expanded.
□ We wish to thank the reviewer for this suggestion and re-emphasize that effective crisis management of COVID-19 pandemic has been essential in navigating the complexities of the

situation and coordinating responses across various sectors. As a result of reviewer's comment, we have expanded the introduction section by adding the following paragraph on the crisis management in the global framework of our paper:

Situational crisis management in the context of COVID-19 involved dynamic assessments of the evolving situation, adaptation to changing circumstances, and coordinated actions among diverse stakeholders. Within this framework, patterns of collaborative actions have emerged, guided by interactionist theories, aimed at achieving expected outcomes while navigating unstructured collaborative processes. Interactionist theories emphasize the dynamic interplay of individuals, groups, and organizations in shaping collective behavior. In the context of COVID-19 crisis management, these theories highlight the importance of social interactions, shared understandings, and emergent behaviors in driving collaborative responses. Rather than relying solely on hierarchical structures, interactionist perspectives recognize the need for flexible, adaptive approaches that allow for decentralized decision-making and emergent solutions. Patterns of collaborative actions in managing a large scope crisis such as the COVID-19 involve multi-level coordination among government agencies, healthcare organizations, businesses, community groups, and individuals. These collaborative efforts encompass a range of activities, including information sharing, resource allocation, policy development, public communication, and implementation of preventive measures. Expected outcomes should be the containment of virus spread, reduction of healthcare burden and protection of vulnerable populations.

However, the COVID-19 crisis has also underscored the challenges of navigating unstructured collaborative processes, characterized by ambiguity, conflicting priorities, and decentralized decision-making. Interactionist theories offer insights into how individuals and groups negotiate meaning, establish social norms, and adapt to changing circumstances within these collaborative contexts. Flexible, adaptive approaches that emphasize communication, trust-building, and shared goals are essential for effective collaboration amidst uncertainty. Along these lines, Awareness Information Network (AIN) approach serves as a platform for facilitating collaboration and information exchange among stakeholders involved in crisis management. This network enables real-time communication, data sharing, and coordination of response efforts across organizational boundaries. Semiotic validation plays a crucial role in ensuring the clarity, consistency, and effectiveness of communication within the AIN, minimizing misinterpretation and fostering trust among stakeholders. In sum, effective crisis management in the context of a large intensity crisis such as the COVID-19, requires a dynamic, collaborative approach informed by interactionist theories. By leveraging patterns of collaborative actions, navigating unstructured collaborative processes, and can utilize tools such as the Awareness Information Network [Daneshgar F., Chattopadhyay S. –"A Framework for Crisis Management in Developing Countries". Intelligent Decision Technologies: an international journal (2011); 5(2): pp. 189-199. DOI: 10.3233/IDT-2011-0106. and Ray P., Chattopadhyay S. – "Fuzzy Awareness Model for Disaster Situations". Intelligent Decision Technologies: an international journal [Special Issue on Intelligent Decision Making in Dynamic Environments: Methods, Architectures and Applications] (2009); 3 (1): pp. 75-82.].

Institut Européen des Membranes,
CNRS et Université de Montpellier
300 Av. du Professeur Emile Jeanbrau
34090 Montpellier, France

3/ Besides, compare the proposed methodology with the latest papers.
 We thank the reviewer for this suggestion. To address this comment, we have added to the manuscript the following section:
Recent literature on spatially resolved Covid19 epidemiology mostly focuses on simulating the spread the disease in time at the scale on countries such as in Parisi et al. [Parisi A, Brand SPC, Hilton J, Aziza R, Keeling MJ, Nokes DJ. Spatially resolved simulations of the spread of COVID-19 in three European countries. PLoS Comput. Biol. 2021 Jul 20;17(7):e1009090. doi: 10.1371/journal.pcbi.1009090. PMID: 34283832; PMCID: PMC8323901 and O. Thomine, S. Alizon, C. Boennec, M. Barthelemy. M. T. Sofonea (2021) Emerging dynamics from high-resolution spatial numerical epidemics eLife 10:e71417. https://doi.org/10.7554/eLife.71417] or at the scale of counties such as in [M. Oliver, D. Georges, C. Prieur, Spatialized epidemiological forecasting applied to Covid-19 pandemic at departmental scale in France, Systems & Control Letters, Volume 164, 2022, 105240, https://doi.org/10.1016/j.sysconle.2022.105240.] using Suspected/Infected/Recovered (SIR) differential coupled equations approaches. To our knowledge, the work introduced in this manuscript offers a unique and first of its kind perspective regarding spatially resolved epidemiology at the localized scale of urban geography at the zip code level while incorporating physical urban descriptors and parameters.

Reviewer #2: I recommend the authors to resubmit the paper after addressing the internal comments and discussing other models.

1/ I see the manuscript is still under construction with several comments within the MS.
 We would like to apologize to the reviewers for having left commented sections in the manuscript, which at the time was still in the final stages of editing. We have corrected this issue and removed comment sections and tracked changes from the revised and herein submitted manuscript.

2/ I recommend to enrich the literature review with other models. Some are cited below which I recommend authors to cite in their paper.
 We wish to thank the reviewer for this suggestion and to enrich the introduction section of the manuscript we expanded on the topic of Covid-19 crisis management and cited additional and proposed references:

Situational crisis management in the context of COVID-19 involved dynamic assessments of the evolving situation, adaptation to changing circumstances, and coordinated actions among diverse stakeholders. Within this framework, patterns of collaborative actions have emerged, guided by interactionist theories, aimed at achieving expected outcomes while navigating unstructured collaborative processes. Interactionist theories emphasize the dynamic interplay of individuals, groups, and organizations in shaping collective behavior. In the context of COVID-19 crisis management, these theories highlight the importance of social interactions, shared understandings, and emergent behaviors in driving collaborative responses. Rather than relying solely on hierarchical structures, interactionist perspectives

recognize the need for flexible, adaptive approaches that allow for decentralized decision-making and emergent solutions. Patterns of collaborative actions in managing a large scope crisis such as the COVID-19 involve multi-level coordination among government agencies, healthcare organizations, businesses, community groups, and individuals. These collaborative efforts encompass a range of activities, including information sharing, resource allocation, policy development, public communication, and implementation of preventive measures. Expected outcomes should be the containment of virus spread, reduction of healthcare burden and protection of vulnerable populations.

However, the COVID-19 crisis has also underscored the challenges of navigating unstructured collaborative processes, characterized by ambiguity, conflicting priorities, and decentralized decision-making. Interactionist theories offer insights into how individuals and groups negotiate meaning, establish social norms, and adapt to changing circumstances within these collaborative contexts. Flexible, adaptive approaches that emphasize communication, trust-building, and shared goals are essential for effective collaboration amidst uncertainty. Along these lines, Awareness Information Network (AIN) approach serves as a platform for facilitating collaboration and information exchange among stakeholders involved in crisis management. This network enables real-time communication, data sharing, and coordination of response efforts across organizational boundaries. Semiotic validation plays a crucial role in ensuring the clarity, consistency, and effectiveness of communication within the AIN, minimizing misinterpretation and fostering trust among stakeholders. In sum, effective crisis management in the context of a large intensity crisis such as the COVID-19, requires a dynamic, collaborative approach informed by interactionist theories. By leveraging patterns of collaborative actions, navigating unstructured collaborative processes, and can utilize tools such as the Awareness Information Network [Daneshgar F., Chattopadhyay S. –"A Framework for Crisis Management in Developing Countries". Intelligent Decision Technologies: an international journal (2011); 5(2): pp. 189-199. DOI: 10.3233/IDT-2011-0106. and Ray P., Chattopadhyay S. – "Fuzzy Awareness Model for Disaster Situations". Intelligent Decision Technologies: an international journal [Special Issue on Intelligent Decision Making in Dynamic Environments: Methods, Architectures and Applications] (2009); 3 (1): pp. 75-82.].

Reviewer #3: This paper presents a probabilistic model to provide an accurate predictive tool for the COVID 19 regardless of the geographical location. The results show that although disease screening and vaccination policies to containment and lockdowns remain critical in controlling the spread of airborne diseases, urban factors should also be taken into consideration when identifying resources and planning targeted responses to mitigate the impact and severity of the virus transmitted through air. Overall, this paper is well-written and easy to follow. The motivation and modelling methods are quite solid, despite having few concerns, noted below. Especially, I appreciate the authors' effort on the inline comments.

1/ Urban factors: the proposed probabilistic CFR models are mainly based on 3 distinctive urban factors: personal, indoor and outdoor. I am wondering how these factors are decided? It would be nice if there are some supporting evidences from existing research?

→ We wish to thank the reviewer for this comment and would like to offer the following explanation. The three distinctive urban factors: personal, indoor and outdoor, were introduced to the methodology after a careful and extensive literature review based on our experience in urban physics and modeling urban climate. The components of each factor were evaluated by using additional physical parameters. The outdoor factor was derived using the built-up density, a disorder quantifier of the city texture and relative humidity, showing that only one dominates. The indoor factor is made of the number/residential unit and residential units/building ratios both of which offer significant contribution to the derived factor. The personal factor in the end reduced to the fraction of population older than 65 years old once we realized that wealth parameter did not have a meaningful statistical contribution and that the access to healthcare was extremely correlated to the fraction of population older than 65 years old. This set of only 5 parameters capture all possible levels of sources of severity determinants: individual, household/sphere of interaction, community/population. All relevant papers are cited in the manuscript to support this methodology of deriving three urban parameters.

2/ Practical implication: While the authors have given a thorough discussion of the model effectiveness, will it have any practical implications regarding the real-world policy making?
 We thank the reviewer for raising this question and would like to provide reassurance that it is indeed our intention and hope that that local authorities in charge of healthcare management can use the findings from our work in the case of an extreme intensity crisis such as the Covid19 pandemic, to tailor health responses and reduce the burden on not only the healthcare but also the urban community. To elaborate more on this topic and protection of vulnerable population, we added the following section to the manuscript:

Amid the COVID-19 pandemic, effective crisis management has been essential in navigating the complexities of the situation and coordinating responses across various sectors. Situational crisis management in the context of COVID-19 involved dynamic assessments of the evolving situation, adaptation to changing circumstances, and coordinated actions among diverse stakeholders. Within this framework, patterns of collaborative actions have emerged, guided by interactionist theories, aimed at achieving expected outcomes while navigating unstructured collaborative processes. Interactionist theories emphasize the dynamic interplay of individuals, groups, and organizations in shaping collective behavior. In the context of COVID-19 crisis management, these theories highlight the importance of social interactions, shared understandings, and emergent behaviors in driving collaborative responses. Rather than relying solely on hierarchical structures, interactionist perspectives recognize the need for flexible, adaptive approaches that allow for decentralized decision-making and emergent solutions. Patterns of collaborative actions in managing a large scope crisis such as the COVID-19 involve multi-level coordination among government agencies, healthcare organizations, businesses, community groups, and individuals. These collaborative efforts encompass a range of activities, including information sharing, resource allocation, policy development, public communication, and implementation of preventive measures. Expected outcomes should be the containment of virus spread, reduction of healthcare burden and protection of vulnerable populations.

Institut Européen des Membranes,
CNRS et Université de Montpellier
300 Av. du Professeur Emile Jeanbrau
34090 Montpellier, France

However, the COVID-19 crisis has also underscored the challenges of navigating unstructured collaborative processes, characterized by ambiguity, conflicting priorities, and decentralized decision-making. Interactionist theories offer insights into how individuals and groups negotiate meaning, establish social norms, and adapt to changing circumstances within these collaborative contexts. Flexible, adaptive approaches that emphasize communication, trust-building, and shared goals are essential for effective collaboration amidst uncertainty. Along these lines, Awareness Information Network (AIN) approach serves as a platform for facilitating collaboration and information exchange among stakeholders involved in crisis management. This network enables real-time communication, data sharing, and coordination of response efforts across organizational boundaries. Semiotic validation plays a crucial role in ensuring the clarity, consistency, and effectiveness of communication within the AIN, minimizing misinterpretation and fostering trust among stakeholders. In sum, effective crisis management in the context of a large intensity crisis such as the COVID-19, requires a dynamic, collaborative approach informed by interactionist theories. By leveraging patterns of collaborative actions, navigating unstructured collaborative processes, and can utilize tools such as the Awareness Information Network.

Institut Européen des Membranes,
CNRS et Université de Montpellier
300 Av. du Professeur Emile Jeanbrau
34090 Montpellier, France
