# Supplementary material for: Urban environmental and population factors as determinants of COVID-19 severity: A spatially-resolved probabilistic modeling approach
Source: PLOS Digit Health. 2025 Jul 18;4(7):e0000921. doi: 10.1371/journal.pdig.0000921 (PMC12274012; doi:10.1371/journal.pdig.0000921)
Supplement: Attachment — Submitted filename: Review Report - PDIG-D-23-00487R1.pdf [file pdig.0000921.s018.pdf]

I appreciate the authors' efforts in addressing previous feedback. While progress has been made, there are still areas for further enhancement in several areas. Below are detailed comments and recommendations aimed at improving the overall quality and impact of the manuscript:

Organization:

1. **Structure Order:**
    - The paper's current structure places the Materials and Methods section at the end, which is unconventional. Typically, it should follow the Introduction to provide readers with context before detailing the methodology. Reorganize the manuscript to: Introduction, Materials and Methods, Results, Discussion, and Conclusion with Future Directions.
2. **Introduction:**
    - The Introduction adequately frames the study's context and importance. However, it could benefit from more explicit research questions or hypotheses to guide readers through the paper's objectives.

Materials and Methods:

1. **Clarity and Detail:**
    - While the methodology is described, there are gaps in specific details such as data collection protocols, participant selection criteria, and the tools used for analysis. Provide more clarity on these aspects to ensure reproducibility.
2. **Sequence and Detailing:**
    - Ensure a clear and logical sequence of methods. Describe each step comprehensively, including any statistical methods or software used, to enhance transparency and reproducibility.

Results:

1. **Presentation Clarity:**
    - Results are presented clearly, but ensure each finding is linked back to the research questions or hypotheses posed in the Introduction. This alignment strengthens the narrative flow and relevance of the findings.
2. **Data Transparency:**
    - Consider including supplementary materials with raw data or detailed tables to support the reported results and allow for deeper scrutiny by readers and reviewers.

Discussion:

1. **Interpretation and Contextualization:**
    - The Discussion section should not only summarize the findings but also interpret their implications in the broader context of peer assessment in virtual learning environments during the COVID-19 era. Relate findings to existing literature and theoretical frameworks to strengthen the discussion's depth.
2. **Future Directions:**

- Expand on the implications of the findings for future research or educational practices. Address potential limitations identified in the study and suggest avenues for further exploration to advance the field.

Conclusion:

1. **Summarization:**
   - The Conclusion should succinctly summarize key findings and their implications without introducing new information. This section should reinforce the paper's contributions to the field.

Language and Style:

- Review the manuscript for clarity and coherence. Ensure that complex concepts are explained concisely and that language is accessible to a broad audience of educators and researchers.

References:

- Verify that all references are recent, relevant, and properly formatted according to the journal's guidelines. Ensure consistency in citation style throughout the manuscript.

Reviewer Feedback:

- Address all reviewer comments comprehensively, particularly those related to methodology clarity, theoretical grounding, and implications for practice.
