# Supplementary material for: Urban environmental and population factors as determinants of COVID-19 severity: A spatially-resolved probabilistic modeling approach
Source: PLOS Digit Health. 2025 Jul 18;4(7):e0000921. doi: 10.1371/journal.pdig.0000921 (PMC12274012; doi:10.1371/journal.pdig.0000921)
Supplement: Attachment — Submitted filename: reply_reviewers_Covid_paper - Sept2024 - final.docx [file pdig.0000921.s020.docx]

|  |  |  |
| --- | --- | --- |

Dr. Habil. Roland J.-M. Pellenq

Director of Research with CNRS (DRCEx)

Email: roland.pellenq@cnrs.fr
Tel: (+33) 6 62 92 28 33

Montpellier, September 21^st^ 2024

Dear Editor,

Please here below the reply to the second round of referee’s comments on our manuscript entitled:

*How do Urban Environment and Population Characteristics Control the Severity of COVID-19? Toward spatially-resolved epidemiology*

By

Jacob Roxon^1^, Marie-Sophie Dumont^1,3^, Eric Vilain^1,4^, Mircea T. Sofonea^2^, Roland J.-M. Pellenq^1,5^*

^1^ EpiDaPo Lab - CNRS / George Washington University Children’s National Medical Center, Children’s Research Institute, 111 Michigan Ave. NW, Washington, DC 20010, USA

^2^ Pathogenesis and Control of Chronic and Emerging Infections (PCCEI, U1058) – University of Montpellier, INSERM, EFS, Univ. Antilles and CHU de Nimes, France

^3^ iGLOBES laboratory, CNRS, ENS-Paris/PSL Université and the University of Arizona – Marshall Building 845 N. Park Avenue - PO Box, 210158-B - Tucson AZ, USA

^4^ School of Medicine, 1001 Health Sciences Road, Irvine CA, USA

^5^ Institut Européen des Membranes, CNRS and University of Montpellier, 300 Avenue du Professeur Jeanbrau, 34090, Montpellier, France

* Corresponding author: Roland J.M. Pellenq.

Email: roland.pellenq@cnrs.fr

Yours sincerely

Dr. Roland Pellenq

**Reviewer #1**

**Comment #1:** The paper's current structure places the Materials and Methods section at the end, which is unconventional. Typically, it should follow the Introduction to provide readers with context before detailing the methodology. Reorganize the manuscript to: Introduction, Materials and Methods, Results, Discussion, and Conclusion with Future Directions.

**🡺** We thank the reviewer for her/his suggestions to comply with PLOS DH requirements. The manuscript’s structure has been modified to Introduction, Materials and Methods, Results, Discussion, and Conclusion in that order.

**Comment #2 :** The Introduction adequately frames the study's context and importance. However, it could benefit from more explicit research questions or hypotheses to guide readers through the paper's objectives.

**🡺** The following text was added to the first part of the introduction section:

*“Ergo, ability to predict CFR not only is a crucial metric in predicting emergency and health care deployment, but it also becomes a viable tool to control more effectively the spread of airborne diseases, including COVID-19, with potential of their accurate determination. However, variables that contribute to CFR are not clearly understood, hence leaving an opportunity for improvement, which is the premise of this study.”*

**Comment #3 :** While the methodology is described, there are gaps in specific details such as data collection protocols, participant selection criteria, and the tools used for analysis. Provide more clarity on these aspects to ensure reproducibility.

**🡺** We thank the reviewer for this comment as we are dedicated to make our paper used by others**.** The Materials and Methods section together with the SI section provide all needed details regarding the selection and analysis processes. In order to improve the clarity and reproducibility of our work, we added the following sections to the manuscript:

*“Our first data selection criterion was formulated with the availability of daily COVID-19 data, reported cases of deaths and infections, both of which are necessary to derive CFR (Eq. 1, SI).* *For the selection time period, all daily values must have been present in the data report in order to be accepted in this study.”*

🡺 We also added the following section to SI:

*“Missing or incomplete data for one of the groups meant that we couldn’t use data in our analysis. The only exception were three cities in France, where COVID-19 data was not publicly available, but due to availability of other inputs, we used cities in France to exemplify the impact of the results of our study. For the analysis of data and statistical modeling a standard spreadsheet editing software package provides adequate capabilities to clean and analyze data, which in the context of this study was Microsoft Office Excel. For mapping of data, we resorted to using open source QGIS software, but most standard geographical information system (GIS) software package could be used for reproducibility.”*

**Comment #4:** Ensure a clear and logical sequence of methods. Describe each step comprehensively, including any statistical methods or software used, to enhance transparency and reproducibility.

**🡺** We thank the reviewer for the asking us to improve the sequence of used methods: the sequence of steps in the methods sections has been adjusted in the manuscript. In addition, the following section in the SI was added:

*“For the analysis of data and statistical modeling a standard spreadsheet editing software package provides adequate capabilities to clean and analyze data, which in the context of this study was Microsoft Office Excel. For mapping of data, we resorted to using open source QGIS software, but most standard geographical information system (GIS) software package could be used for reproducibility.”*

🡺 We also added this paragraph to the manuscript:

*“The model weight parameters, w’s, have been optimized by minimizing the absolute sum of errors between predicted and measured CFR(λ_1 ) values using a non-linear optimization regression model with the outcome values restricted to fall between 0 and 1. Because the CFR and a_i values span over several orders of magnitudes, to minimize the impact of any potential outliers in the regression analysis, a sum of absolute errors, ε, approach has been utilized”*

**Comment #5:** Results are presented clearly, but ensure each finding is linked back to the research questions or hypotheses posed in the Introduction. This alignment strengthens the narrative flow and relevance of the findings.

**🡺** In order to make the result easier to understand and follow in connection to the main questions being addressed in this work, in the results section, we added the following sentence:

*“Consequently, offering a tool for measuring the impact that urban factors have on the spread of COVID-19 and proving their specific contributions offering a predictive model, which can be applied to other airborne diseases.”*

🡺 And at the end of the sections [for cities taken as a whole, this ratio remains unity]*, we added the following sentence: “showing the statistical significance of the model established in this study to address the question of what. variables contribute to CFR and their impact.”*

**Comment #6**: Consider including supplementary materials with raw data or detailed tables to support the reported results and allow for deeper scrutiny by readers and reviewers.

**🡺** We thank the reviewer for this important suggestion that strengthens our work. Multiple sources of data were used in this study and accessibility to raw data is now clearly presented in the SI and listed in tables S1-3 and S8.

**Comment #7:** The Discussion section should not only summarize the findings but also interpret their implications in the broader context of peer assessment in virtual learning environments during the COVID-19 era. Relate findings to existing literature and theoretical frameworks to strengthen the discussion's depth.

**🡺** This is an interesting comment: in the broader context to comment on the impact of virtual learning, the following sentences were added to discussion section: *“However, with the shift toward more remote work and learning environments, we would expect a shift in the specific contribution of indoor factor variables, which in the context of this study for the initial period of COVID-19, studied regions were not affected by any restrictive social distancing or confinement policies. This would ultimately replicate the impact of social distancing norms implemented in cities categorized in this study as outliers, which would reduce CFR values.”*

**Comment #8**: Expand on the implications of the findings for future research or educational practices. Address potential limitations identified in the study and suggest avenues for further exploration to advance the field.

**🡺** The following section was added to the final part of the discussion section:

*”As a validation of our approach, the a_0 of community-acquired pneumonia is very close to that of COVID-19 first wave while that of successive COVID-19 waves is converging to the a_0 of influenza (i.e. 10 times less that of pneumonia of COVID-19(λ1), which offers an opportunity for further improvements and expansion of this study, that is evaluation of other airborne diseases such as influenza, especially at the localized scale of zip codes. This would provide a path for more comprehensive geo-spatial analysis on the quality of healthcare together with accessibility to local health and treatment centers which in this study were assumed to be uniformly distributed throughout a specific geo-location and thus having a fixed impact on the CFR. In addition, more localized approach will offer more accurate insights on the impact of remote learning and work environments, which without any specific city-wide seasonal disease responses, may vary throughout different parts of cities, ranging from predominately residential neighborhoods to more congested industrial and commercial regions with higher traffic densities.”*

**Comment #9:** The Conclusion should succinctly summarize key findings and their implications without introducing new information. This section should reinforce the paper's contributions to the field.

**🡺**We thank the reviewer for this suggestion that improves the conclusion section. We added a conclusion section with the following information:

*“In this study, we analyzed the first wave of COVID-19 data measured through CFR for 118 locations (city zip-codes, city boroughs, and cities) worldwide and found a strong correlation between CFR and urban parameters, uniquely classified as outdoor, indoor and personal factors. We showed that the derived probabilistic model provides accurate predictions regardless of the geographical location. It also scales to other infectious diseases such as flu and pneumonia suggesting that when adjusted for the population, herein proposed approach can be used to evaluate risk and severity of airborne diseases at different geo-spatial levels and provides a confirmation that urban factors such as population density, humidity, or order of buildings, should be considered when identifying resources and planning targeted responses to mitigate the impact and severity of the viruses transmitted through air.”*

**Comment #10:** Review the manuscript for clarity and coherence. Ensure that complex concepts are explained concisely and that language is accessible to a broad audience of educators and researchers.

**🡺** The manuscript has been reviewed for any errors and coherence. Minor changes in text have been implemented.

**Comment #11:** Verify that all references are recent, relevant, and properly formatted according to the journal's guidelines. Ensure consistency in citation style throughout the manuscript.

🡺 The list of references is accurate and represents a great deal of published and most relevant work regarding the COVID19 2020-2021 period. Actually, these were the source of our inspiration to derive this proposed probabilistic approach leading to the simple a_i equation that contains only statistical weights w’s values that characterize city geometry and texture, local climate, and population. As proofs of the relevance of this equation, is the fact that these w’s parameters are able to perform (i) a multiscale of the geo-localization of COVID19 intensity from the zip code level to the global city scale (and in counties where urban population dominates) (ii) accurate predictions for flu and pneumonia with no adjustment of the w’s values (hence assuming constant city texture and geometry, local urban climate and population profile, which is correct at the considered scale of time windows). This led us to conclude that COVID19 pristine intensity in the considered period of time (hence variants, with no extensive vaccination scheme in place) was indeed 10 times that of flu and equivalent to that of pneumonia further confirming on-the-ground measurements.

**Reviewer #4:**

**Comment #1**: Model fitting and optimization: The authors did not provide sufficient details on the convergence criteria, starting values, and sensitivity of the optimization process. The optimization was performed on a relatively small dataset, which may not be sufficient to robustly estimate the model parameters.

🡺 The robustness of the approach was thoroughly checked in this paper. Indeed, the same equation with the same w’s values) gives a_0 values (the pristine severity) of the considered diseases (COVID19, flu, pneumonia) that are in full agreement with field measurements at the scale of cities. Furthermore, focusing on the COVID19 results, we demonstrate that our approach is capable of a multi-scale assessment of the severity of the disease from the zip code level to the global city scale (and county and country scales where urban population dominates). From a mathematical point of view, it is very reinsuring to find that the equation performs well with constant w’s that indeed only marginally change overtime (city geometry and texture, urban climate, population profile) whatever the 3 considered airborne diseases. Data from the 18 neighborhoods and boroughs from 4 major US cities were used to determine statistical weights for all the 5 w’s and the a_0 CFR parameter, which formed a statistical problem of 6 unknown variables and 18 data points. With a non-linear fitting procedure, we minimized the sum of the absolute value of the difference between the predicted and the actual data for 18 data points (boroughs) in an over-deterministic setup, which meant more data points than unknowns. Our approach given its multi-scales and multi-diseases performances is shown to be very robust. However, it can be made more accurate by adding new physical quantities with their corresponding statistical weights to be determined, if available data is available.

**Comment #2:** Model validation and goodness-of-fit: The authors used limited statistical metrics (R^2 and MAE) to assess the model's performance, and did not report any formal statistical tests to evaluate the significance of the model fit or the individual urban factor contributions.
**🡺** As shown in Figure 3 of the manuscript, we checked that Outdoor, Indoor and Personal parameters follow a gaussian distribution, hence offering confidence in the strength of their individual contributions to the model. It is found that the Indoor criterion (composed on the number of housing units per building and number of dwellers per units) is the dominating factor for the severity of the COVID19 disease, which can be explained by higher contamination rates in common spaces (corridors, elevators, staircases in large building systems) due to stability of virus-laden mucus droplets sneezed out by contaminated people. Interestingly, we found that in the outdoor factor, the city texture represented by the local order parameter, which quantifies the geometrical order of local buildings that impact air flow, accounts for only 12% while the relative air humidity (88%) and zero contribution to the built density. It has been well accepted that virus mechanical stability of its corona-bilipid envelop is linked to relative humidity and the level of evaporation of laden mucus droplets (see Poon et al, Soft matter science and the COVID-19 pandemic, DOI: 10.1039/D0SM01223H, Soft Matter, 2020, 16, 8310-8324 for instance). Interestingly the personal factor can be reduced to one component, which is a fraction of urban dwellers older than 65. Finally in the validation process of the overall approach it was assuring to find that the a_0 parameter for COVID19 (first wave) disease was close to that of pneumonia and 10 times larger than that of the flu (contrary to the unscientifically grounded public opinions and posture of some media outlets across the world).

**Comment #3 part1:** Assumptions and limitations of the statistical approach: The authors did not address potential issues of multicollinearity or interdependence among the urban factors,

🡺 We thank the reviewer for this comment that is essential. The a_i equation is built as the product of 3 independent probabilities (referred to as outdoor, indoor and personal factors throughout the paper). Each of these is composed of 1 to 3 components which have to sum up to unity by construction. The choice of which component to choose was driven from the relevant cited literature. However, these have to be independent quantities, an example being relative humidity and urban texture order parameters, which show no correlation. On the contrary, when these are not independent, such as the fraction of the population and the dwellers revenues, it was reassuring to see that the fitting procedures diminished contribution of one of these values to zero. It is also the case of the urban texture order parameter and the fraction of built lands with respect to the total city area (it was shown that the number of building neighbors to a given building and calculated from the building-building positions two-point correlation function called g(r), actually correlated to the urban order parameter in a decreasing exponential form: the larger the number of neighbors, the smaller the urban texture order parameter). The overall fitting procedure hence left 5 statistical weight parameters and the a_0, the pristine CFR severity of the disease as if people were not packed in cities and younger than 65 yo. The fact that the a_0 value for the COVID-19, the flu and pneumonia were in agreement with experiments and that the w’s parameters could be kept constant through city scales (from zip-code to the global urban scale) and also for the different considered diseases, demonstrate the robustness and the soundness of our approach.

**Comment #3 part 2:** nor did they consider potential spatial or temporal autocorrelation in the data.

**🡺** We respectfully wish to indicate that the question spatial or temporal self-correlations among the considered input data out of 18 neighborhoods and boroughs of 4 major cities across the US is simply not relevant as none of the 5 considered weight parameters are local meaning that the 18 locations data points used to determine them are independent.

**Comment #4:** Handling of outliers and missing data: The authors excluded 9 cities from the analysis without a clear justification, and did not address how they handled missing data or outliers in the dataset.

**🡺** We thank the reviewer to rise this point that indeed needs clarification: These refereed 9 cities correspond to municipalities that are either independent territories (such as Maldives islands) or more important agglomerations (such as Berlin, Germany) that took immediate lockdown measures during COVID19 wave-1. Hence the COVID19 intensity they experienced was not reinforced by dwellers movements leading to over-contamination in some urban parts of a city. To address this comment, we added the following sentences to the Methods and Materials section: *“Our first data selection criterion was formulated with the availability of daily COVID-19 data, reported cases of deaths and infections, both of which are necessary to derive CFR (Eq. 1, SI). For the selection time period, all daily values must have been present in the data report in order to be accepted in this study.”*

🡺 We also added the following part: *“Ability to obtain the necessary input data to derive urban factors created the second selection data criterion, namely if there was missing or incomplete input data for urban factors, with the exception of input data for Outdoor and Indoor factors, we chose to neglect such CFR point from our analysis. For Outdoor and Indoor missing data was acceptable for as long as it was within 5% of the temporal scale.”*

There was already an explanation in the manuscript about outliers in the Discussion section:

*It is also important to note that the model significantly overestimated CFR(λ_1 ) for 9 cities (Fig. 4a, Table S5) where measured CFR(λ_1 ) were very close to a_0 (λ_1 ). Upon further investigation, these cities were found to be either isolated territories (such as Maldives or Malta, which, based on size and population, are treated as cities) or quick to adopt social distancing and/or lockdown measures (as in San Francisco CA, Berlin in Germany, or Singapore) during the first wave, λ_1, of the COVID-19 pandemic. We can conclude therefore that measured CFR as predicted by the coronavirus severity equation (Eq.2) reflects the absence or inefficiency of sanitary measures taken by local and/or national authorities to manage the first wave of COVID-19.”*

However, to make a stronger point about outliers, we also modified explanation about the strength of correlation in Fig.4b to include information about outliers:

*“It is shown that the approach remains operative at the global city scale making model fit predictions with a high coefficient of determination, R^2=0.95 and a low RMSE=0.31 for population adjusted CFR_pop after excluding 9 cities, which were the only ones in the sample size to facilitate timely deployment of targeted countermeasures and confinement strategies during the first wave of the COVID-19 pandemic* *or were identified as remote locations.”*

**Comment #5:** Generalizability and transferability of the model: The authors did not provide a clear assessment of the model's transferability to other geographical contexts or its ability to predict CFR during different stages of the pandemic.

**🡺**This is an important comment and we wish to thank the reviewer for raising it. In the discussion section, we have already addressed the question about transferability of the model, by adjusting a_0 only (i.e. specific to a given disease keeping all w’s constant). Specifically:

*“we could solely adjust a_0 (λ_i ) to a_0 (λ_3 )= 0.002 and a_0 (λ_4 )=0.001 respectively while maintaining acceptable accuracy of predictions. The sharp decline in a_0 (λ_i ) values over time reflects the immense effect of the vaccine campaign on the severity of the pandemic and the transition to virus variants being less harmful in terms of severe cases (i.e. deaths). The variability in a_0 (λ_i ) is expected as over the course of the entire pandemic, with yearly average H_R values, city layouts and census data exposed to minimal changes, w weight parameters should not vary to a noticeable extent”*

Regarding geographical transferability, the sample data used in this study has 30 cities from across the world, 28 neighborhoods and 60 zip codes, which provides a comprehensive (the largest to date within a study of this kind) multi-spatial and geographical sample to provide evidence for geographical transferability.

**Comment #6:** Interpretation and implications: The study did not provide a clear discussion of the practical implications of the model for public health decision-making and urban planning.

**🡺** To address this concern, the following part was added to the discussion section:

*“Furthermore, with wider adoption of IoT systems and improvements in data sharing capabilities with next generation wireless technologies, this modeling can be utilized to identify more effectively and efficiently risky or hot spots during viral seasons. In the context of urban planning, leveraging data of this kind, can help city planners and architects optimize development of buildings, blocks and cities to optimize urban factors to provide lowest possible CFR values.”*

**Comment #7:** Ethical and equity considerations: The study did not address potential ethical or equity concerns related to the use of urban factors to predict disease severity and target interventions.
**🡺** This is an interesting point and we thank the reviewer to point this out. First, we have used only publicly available data regarding city geometry, texture, local climate, urban design and population profile. Regarding COVID19 data, we also used publicly available data from municipalities websites. This approach is therefore subjected to the quality of the data reporting and scale (zip codes, boroughs…): we found in the course of this work that US, Indian, Brazilian cities were constantly providing a reliable source of information by contrast to French ones where the smaller scale of aggregated COVID19 data (number of cases and deaths) was the county-scale. The global aim of this work is to provide a frame for a more efficient response regarding infectious airborne disease such as COVID19 ; the risk mapping provides a clear way to allocate resources more efficiently, ICU beds for instance hence providing more equity in the treatment of the disease.

**Comment #8:** The methods section could be improved by providing more details on the model fitting and optimization process, as well as the handling of outliers and missing data.
🡺This has been addressed with answers to comment #4 from reviewer #1 and comments #4 and #5 from the present reviewer (see above).

We summarize the reply to reviewer#1 on this specific point here below along with text modifications: the sequence of steps in the methods sections has been adjusted in the manuscript. In addition, the following section in the SI was added:

*“For the analysis of data and statistical modeling a standard spreadsheet editing software package provides adequate capabilities to clean and analyze data, which in the context of this study was Microsoft Office Excel. For mapping of data, we resorted to using open source QGIS software, but most standard geographical information system (GIS) software package could be used for reproducibility.”*

🡺 We also added this paragraph to the manuscript:

*“The model weight parameters, w’s, have been optimized by minimizing the absolute sum of errors between predicted and measured CFR(λ_1 ) values using a non-linear optimization regression model with the outcome values restricted to fall between 0 and 1. Because the CFR and a_i values span over several orders of magnitudes, to minimize the impact of any potential outliers in the regression analysis, a sum of absolute errors, ε, approach has been utilized”*

**Comment #9:** The study design, using a combination of publicly available data and a novel modeling approach, is generally appropriate for the research question. However, the limitations of the data and the statistical approach need to be better addressed.

🡺 We used a reliable set of available data for urban environment and dwellers descriptions as well as for the COVID19 disease (please see the reply to your comment#7 here above).

**Comment #10:** The statistical analyses have some limitations, as outlined in the major issues section. The authors should consider addressing these limitations to strengthen the reliability and robustness of the findings.

🡺 We respectfully disagree with the reviewer on this point. As already stated in the reply to comments #1 to #5, the robustness of the approach (using the same equation with constant w’s values) give a_0 values (the pristine severity) of the considered diseases (COVID19, flu, pneumonia) that are in comprehensive and full agreement with field measurements. Furthermore, focusing on the COVID19 results, we demonstrated that our approach is capable of a multiscale assessment form zip code level to the global city scale (and probably county and country scales where urban population dominates).

Data from the 18 neighborhoods and boroughs from 4 major US cities were used to determine statistical weights for all the 5 w’s and the a_0 CFR parameter, which formed a statistical problem of 6 unknown variables and 18 data points. With a non-linear fitting procedure, we minimized the sum of the absolute value of the difference between the predicted and the actual data for 18 data points (boroughs) in an over-deterministic setup, which meant more data points than unknowns. Our approach given its multi-scales and multi-diseases performances is shown to be very robust. However, it can be made more accurate by adding new physical quantities with their corresponding statistical weights to be determined, if available data is available.

**Comment #11:** The results generally support the conclusions, but the limitations of the model and the data need to be more thoroughly discussed.

🡺 This comment seems redundant to the previous one leading to the same response (see above). However, we do understand the requirement for safety in the model and the used data as discussed earlier. We took an action to address this by making available to the reader all raw data and files: links to all data have already been included in SI, specifically tables S1-S4 and S8.

**Comment #12:** The data used in the study appears to be publicly available, but the authors should provide clear instructions or links for accessing the data.

🡺 Please see the reply to the previous comment regarding all the data used. Links to all data have already been included in SI, specifically tables S1-S4 and S8.

**Reviewer #5**

**Comment #1:** Make sure to elaborate on how the model parameters were derived and discuss their significance in more detail.

🡺 We thank the reviewer to raise his/her concerns regarding the model parameters and their significance. These concerns were also raised by other reviewers which comments (along with our replies) are listed here above. In particular, please see from reviewer #1 comments and responses #3,#4, #11 and from reviewer #4 comments and responses #1, #2, #3, #4, #5, #8, #10.

**Comment #2:** Additionally, consider addressing other potential factors that may influence the Case Fatality Rate (CFR) but were not included in the model, such as healthcare infrastructure and public health policies.

🡺 We thank the reviewer for this important point: Access to health care system and infrastructure and related policies was found to be very strongly correlated to the fraction of population above 65 years old. At the exception of Arabic gulf countries, we found data for 200+nations, showing a strong (almost perfect) linear correlation between yearly health care spending and the 65+ yo population (see SI). Hence this fraction of population represents in a statistical fashion the access of healthcare systems. Along the same line, the dweller income is found to have no statistical weight, which can be explained by the urgency of protecting the population during COVID19 first waves regardless of their individual income levels.
