# Supplementary material for: Urban environmental and population factors as determinants of COVID-19 severity: A spatially-resolved probabilistic modeling approach
Source: PLOS Digit Health. 2025 Jul 18;4(7):e0000921. doi: 10.1371/journal.pdig.0000921 (PMC12274012; doi:10.1371/journal.pdig.0000921)
Supplement: Attachment — Submitted filename: reply_reviewers_Covid_paper_Dec_2024 - 12-31-2024.docx [file pdig.0000921.s021.docx]

|  |  |  |
| --- | --- | --- |

Dr. Habil. Roland J.-M. Pellenq

Director of Research with CNRS (DRCEx)

Email: roland.pellenq@cnrs.fr
Tel: (+33) 6 62 92 28 33

Montpellier, December 11^th^ 2024

Dear Editor,

Please here below the reply to the THIRD round of referee’s comments on our manuscript entitled:

*How do Urban Environment and Population Characteristics Control the Severity of COVID-19? Toward spatially-resolved epidemiology*

By

Jacob Roxon^1^, Marie-Sophie Dumont^1,3^, Eric Vilain^1,4^, Mircea T. Sofonea^2^, Roland J.-M. Pellenq^1,5^*

^1^ EpiDaPo Lab - CNRS / George Washington University Children’s National Medical Center, Children’s Research Institute, 111 Michigan Ave. NW, Washington, DC 20010, USA

^2^ Pathogenesis and Control of Chronic and Emerging Infections (PCCEI, U1058) – University of Montpellier, INSERM, EFS, Univ. Antilles and CHU de Nimes, France

^3^ iGLOBES laboratory, CNRS, ENS-Paris/PSL Université and the University of Arizona – Marshall Building 845 N. Park Avenue - PO Box, 210158-B - Tucson AZ, USA

^4^ School of Medicine, 1001 Health Sciences Road, Irvine CA, USA

^5^ Institut Européen des Membranes, CNRS and University of Montpellier, 300 Avenue du Professeur Jeanbrau, 34090, Montpellier, France

* Corresponding author: Roland J.M. Pellenq.

Email: roland.pellenq@cnrs.fr

Yours sincerely

Dr. Roland Pellenq

**Reviewer #6**

**Comment #1:** The research emphasizes advanced methodology over the practical application of this approach. This focus is valid if the primary aim is to showcase the methodology, with results playing a secondary role. However, if the findings are intended to be the central focus of the article, I would recommend clarifying the direction—either concentrating solely on methodological innovation or prioritizing practical application.

**Response #1:** We thank the reviewer for this very valid comment. This work serves indeed two purposes: (i) the prediction of differentiated Covid19 pandemic intensities at different spatial scales from Zip code level to the global city scale but also in time with the evaluation of the pristine CFR value for different pandemic waves ; all being in agreement with actual field data and (ii) the partial demonstration that **t**he approach can be used for other airborne-transmitted disease such as flu or pneumonia (“partial demonstration” because only we implemented the methodology upon previous reviewer request on only 3 US cities).

**Comment #2:** The research presented is thorough and significant. However, it is challenging to read due to an overwhelming amount of continuous explanations, often requiring multiple readings of the same sentence for clarity. The frequent use of scientific notations, while familiar to the authors, in the text further complicates comprehension. Reducing the integration of notations within explanations and opting for more textual descriptions could improve readability.

**Response #2:** We do thank the reviewer for this point aiming at improving paper’s clarity. To address this comment, glossaries to both the manuscript and SI with a short definition of frequent (manuscript) and all (SI) notations used in this work. We also edited the first section of the introduction section reducing length of sentences.

**Comment #3:** Classifications – social sciences appear twice

**Response #3**: We removed the repetition “social sciences”.

**Comment #4:**

Keywords – covid-19 and corona virus appear together, better to leave the 1st term.

**Response #4:**We replaced “corona virus” term with “Airborne Diseases”.

**Comment #5:** Abstract – instead of the word "link" use connection or association.

**Response #5:** We changed the word "link" to “connection”.

**Comment #6:** The 1st paragraph of the introduction – rephrase, make sentences shorter. You have sentence spanning on 5 rows. In general, use this suggestion for the rest of the text. There are very long sentences. Even sentences that start before presenting the equations and end after the equations. The reading is not smooth.

**Response #6:** We heavily modified the introduction section and made some minor changes to the Materials and Methods section, while also including glossary before the introduction section with most frequent notations to make the manuscript easier to read. Furthermore, we included a full glossary in the supporting information section.

**Comment #7:** Figure 1 – there is only the title of the figure that must be placed under the figure. The comments about tables s1, s2 must by in the text.

**Response #7:** References to supporting materials in the manuscript figure captions’ were removed and placed in the text instead.

**Comment #8:** Was figure 1 prepared specifically for this paper? If not, please provide the reference.

**Response #8:** Figure 1 was prepared specifically for this paper using an open-source software.

**Comment #9:**The literature review is poor. I understand that in some journals there is no need of separate sections of the introduction and literature review, but still there are a lot of statements in the introduction section that require citations.

**Response #9:** We have increased the reference list with the following relevant and very recent works. The following references were added to the Introduction section:

- Shokri A, Mahmoodi H, Piroozi B, Moradi Y, Moradpour F, Moradi G, et al. An assessment of the relationship between national rates of Covid-19 incidence and mortality as reported to an international comparison database: An ecological study. Health Sci Rep. 2023 May;6(5):e1306.

- Mukherjee D, Lamacchia M, DeBari VA. The Interim Case Fatality Rate (ICFR) of the COVID-19 Pandemic for Europe and the United States from March 1, 2020 to August 15, 2021. Does the ICFR Regress to the Final CFR? Ann Clin Lab Sci. 2022 Nov;52(6):863–70.

- Saavedra P, Santana A, Peñate A, Herrera C, Pacheco JM. Evolution of the lethality due to SARS-CoV-2 in Spain according to age group and sex. Sci Rep. 2022 Dec 21;12(1):22052.

- Alzahrani KJ, Sharif N, Khan A, Banjer HJ, Parvez AK, Dey SK. Impact of meteorological factors and population density on COVID-19 pandemic in Saudi Arabia. Saudi Journal of Biological Sciences. 2023 Feb 1;30(2):103545.

- Hossain MP, Zhou W, Leung MYT, Yuan HY. Association of air pollution and weather conditions during infection course with COVID-19 case fatality rate in the United Kingdom. Sci Rep. 2024 Jan 6;14(1):683.

We also added the following references to the Discussion section:

- Martín-Sánchez V, Ruiz-Garcia A, Vitelli-Storelli F, Serrano-Cumplido A, Barquilla-Garcia A, Micó-Pérez RM, et al. Case-fatality rate of SARS-CoV-2 infection during the third and fifth epidemic waves in Spain: Impact of vaccination. Semergen. 2023 Oct;49(7):102026.

- Suwono B, Brandl M, Hecht J, Eckmanns T, Haller S. Epidemiology of healthcare-associated SARS-CoV-2 outbreaks in Germany between March 2020 and May 2022. J Hosp Infect. 2023 Apr;134:108–20.

- Park J, Michels A, Lyu F, Han SY, Wang S. Daily changes in spatial accessibility to ICU beds and their relationship with the case-fatality ratio of COVID-19 in the state of Texas, USA. Appl Geogr. 2023 May;154:102929.

**Comment #10:** Page 4, 2nd paragraph. Is "sane individual" the right term?

**Response #10:** We changed the term to healthy individuals.

**Comment #11:** Page 4, last paragraph. The word "provide" appears twice in the same sentence.

**Response #11:** We remove the repetitive word.

**Comment #12:** The table of all scientific notations should be presented in the materials section.

**Response #12:** We added glossary sections to both the manuscript and supporting information.

**Comment #13:** Page 5. What amount of data that was removed from the analysis?

**Response #13:** We modified part of the Materials and Methods section to provide clarity on this. “If there was any missing or incomplete input data for Personal factor, we chose to neglect such CFR point from our analysis. For Outdoor and Indoor factors, missing data was acceptable for as long as it was within 5% of its temporal scale, i.e. 95% or more of data was present in the sample size used for analysis.”

**Comment #14:** Discussion. The definitions of 3rd and 4th waves are wrong. Each wave couldn't be 1.5-2 years.

**Response #14:** we thank the reviewer for bringing this point to the light. The reference dates were for cumulative vales, however, for clarity and consistency, we provided an actual definition for 3^rd^ and 4^th^ waves used in this study, which is in line with other publications. The revised text of the Discussion section now reads: “With additional cumulative $C{19}_{C}$ and $C{19}_{d}$ data (see SI) between the start of the pandemic and third wave (March 2021 - June 2021) and forth wave (July 2021 - January 2022) respectively, and keeping all w's parameters constant (Fig. 2), we could solely adjust $a_{0}(\lambda_{i})$ to $a_{0}(\lambda_{3})$ = 0.002 and $a_{0}(\lambda_{4})$ = 0.001 respectively while maintaining acceptable accuracy of predictions.”

**Comment #15:** In my opinion, the results part with illustrations must be much more detailed and comparative. The methods part is too detailed.

**Response #15:** We respectfully disagree with the reviewers as all concepts are supported by the minimal theoretical concepts and equations. We did illustrate them with as simple as possible figures. Furthermore, the supporting information section precisely reports all used data and their origin with a web link for each of these so that we did make sure that the reader will be absolutely able to reproduce the results and repeat every single calculation. The SI section also provides more figures that complement the ones in the manuscript. Upon the reviewer suggestion, we have now included glossary sections in both the manuscript and the SI so that the reader has an easy description/definition of the used physical descriptors found in the theoretical part of this work.
