# Supplementary material for: Urban environmental and population factors as determinants of COVID-19 severity: A spatially-resolved probabilistic modeling approach
Source: PLOS Digit Health. 2025 Jul 18;4(7):e0000921. doi: 10.1371/journal.pdig.0000921 (PMC12274012; doi:10.1371/journal.pdig.0000921)
Supplement: Attachment — Submitted filename: reply_reviewers_Covid_paper_May_2025.docx [file pdig.0000921.s022.docx]

|  |  |  |
| --- | --- | --- |

Dr. Roland J.-M. Pellenq

Director of Research with CNRS (DRCEx)

Email: roland.pellenq@cnrs.fr
Tel: (+33) 6 62 92 28 33

Montpellier, May 11^th^, 2025

Dear Editor,

Please here below the reply to the fourth round of referee’s comments on our manuscript entitled:

*Urban Environmental and Population Factors as Determinants of COVID-19 Severity: A Spatially-Resolved Probabilistic Modeling Approach.*

By

Jacob Roxon^1^, Marie-Sophie Dumont^1,3^, Eric Vilain^1,4^, Mircea T. Sofonea^2^, Roland J.-M. Pellenq^1,5^*

^1^ EpiDaPo Lab - CNRS / George Washington University Children’s National Medical Center, Children’s Research Institute, 111 Michigan Ave. NW, Washington, DC 20010, USA

^2^ Pathogenesis and Control of Chronic and Emerging Infections (PCCEI, U1058) – University of Montpellier, INSERM, EFS, Univ. Antilles and CHU de Nimes, France

^3^ iGLOBES laboratory, CNRS, ENS-Paris/PSL Université and the University of Arizona – Marshall Building 845 N. Park Avenue - PO Box, 210158-B - Tucson AZ, USA

^4^ School of Medicine, 1001 Health Sciences Road, Irvine CA, USA

^5^ Institut Européen des Membranes, CNRS and University of Montpellier, 300 Avenue du Professeur Jeanbrau, 34090, Montpellier, France

* Corresponding author: Roland J.M. Pellenq.

Email: roland.pellenq@cnrs.fr

Yours sincerely

Dr. Roland Pellenq

**Comment #1:** Structure Order: The paper's current structure places the Materials and Methods section at the end, which is unconventional. Typically, it should follow the Introduction to provide readers with context before detailing the methodology. Reorganize the manuscript to: Introduction, Materials and Methods, Results, Discussion, and Conclusion with Future Directions.

**Response #1:** The manuscript follows the order of Abstract, Author summary, Introduction, Results (with subsections Materials and Methods and Model Performance), Results, Discussion, and Limitations and Perspectives, Conclusion, Supporting Information, Acknowledgments and References.

Note that we have modified the title to a more appropriate version: Urban Environmental and Population Factors as Determinants of COVID-19 Severity: A Spatially-Resolved Probabilistic Modeling Approach

**Comment #2:** Introduction: The Introduction adequately frames the study's context and importance. However, it could benefit from more explicit research questions or hypotheses to guide readers through the paper's objectives.

**Response #2:** To address this comment, we added to the introduction section the following paragraph:

*To address this gap, our study is guided by the research question of how specific urban environmental factors and population characteristics influence the spatial variability of COVID-19* $CFR$ *at multiple geographic scales and whether a simple probabilistic model incorporating urban factors can accurately predict* $CFR$ *towards targeted public health interventions. To answer this question, we develop and validate a probabilistic model that statistically predicts* $CFR$ *based on three distinctive Urban Factors* *(*$U_{i}$*):* *Personal (*$P_{i}$*), Indoor (*$I_{i}$*), and Outdoor (*$O_{i}$*). We assess the model’s performance across multiple spatial scales and urban contexts, aiming to provide actionable insights for pandemic response and urban health policy. We find that while the approach works best for city-wide predictions (*$N=30$ *cities worldwide), we can also successfully utilize it at other scales, ranging from zip codes or city districts (Fig. S2) to county or even country levels. This approach offers a unique perspective by providing quantitative insights on spatially resolved epidemiology at the localized urban scale, while explicitly including physical parameters and urban descriptors.*

**Comment #3:** Clarity and Detail: While the methodology is described, there are gaps in specific details such as data collection protocols, participant selection criteria, and the tools used for analysis.

Provide more clarity on these aspects to ensure reproducibility.

**Response #3**: Supporting Information section provides detailed methodology to ensure reproducibility and a list describing all SI tables and figures is provided after the conclusion section as requested.

**Comment #4:** Sequence and Detailing: Ensure a clear and logical sequence of methods. Describe each step comprehensively, including any statistical methods or software used, to enhance transparency and reproducibility.

**Response #4:** Supporting Information section provides detailed methodology to ensure reproducibility complementing the essential steps given in the core of the manuscript grounding our theoretical approach.

**Comment #5:** Presentation Clarity: Results are presented clearly, but ensure each finding is linked back to the research questions or hypotheses posed in the Introduction. This alignment strengthens the narrative flow and relevance of the findings.

**Response #5:** The following paragraph was added to the results section: *These findings directly support the objective of this research and show that urban environmental factors, namely personal, indoor and outdoor parameters which encompass age, building and population densities, relative humidity and city texture, not only influence the spatial variability of COVID-19 CFR, but can be used to accurately predict CFR across different urban settings.*

**Comment #6:** Data Transparency: Consider including supplementary materials with raw data or detailed tables to support the reported results and allow for deeper scrutiny by readers and reviewers. **Response #6:** Supporting information includes detailed tables with information on raw data. We also provides in SI tables 1- 4 all needed information (with links to the their actual web location) as they all are publicly available data.

**Comment #7:** Interpretation and Contextualization: The Discussion section should not only summarize the findings but also interpret their implications in the broader context of peer assessment in virtual learning environments during the COVID-19 era. Relate findings to existing literature and theoretical frameworks to strengthen the discussion's depth.

**Response #7:** The following paragraph was added to the discussion section:

*While our primary focus is epidemiological, the insights from our spatially resolved analysis of COVID-19 severity have important implications for peer assessment practices also in virtual learning environments, which became central during the pandemic. The pandemic’s disruption of traditional classroom settings necessitated rapid adoption of online platforms, where peer assessment has emerged as a critical component of collaborative learning. Just as effective crisis management during COVID-19 required adaptive, decentralized, and communicative approaches, as highlighted by interactionist theories and the Awareness Information Network, successful peer assessment in online settings depends on flexible structures that support real-time feedback, trust-building, and shared understanding among students. The variability in COVID-19 outcomes across urban contexts, shaped by factors such as ventilation, density, and social norms, mirrors the disparities observed in student participation and learning outcomes in virtual environments-often influenced by digital access, communication clarity, and group dynamics. As a result, our educators and instructional designers can better structure peer assessment systems that are resilient, equitable, and responsive to the diverse needs of learners, especially in times of crisis and rapid change. This work thus contributes to a broader understanding of how principles from public health crisis management can inform the design and facilitation of effective peer assessment and collaborative learning in virtual educational settings.*

**Comment #8:** Future Directions: Expand on the implications of the findings for future research or educational practices. Address potential limitations identified in the study and suggest avenues for further exploration to advance the field.

**Response #8:** The following paragraph was added to the discussion section:

*To advance the work in the field, future studies should pursue research to directly measure how environmental and social factors influence peer assessment and collaborative learning outcomes in virtual classrooms, explore how socio-economic dynamics evolve over time and across different cultural enivornments while leveraging artificial intelligence to monitor, predict, and support epidemiological surveillance systems.*

**Comment #9:** Summarization: The Conclusion should succinctly summarize key findings and their implications without introducing new information. This section should reinforce the paper's contributions to the field.

**Response #9:** The Conclusion section was restructured to read:

*In this study, we conducted a comprehensive analysis of COVID-19 severity during the first wave of the pandemic, examining Case Fatality Ratio data across 118 locations spanning city zip codes, boroughs, and major cities worldwide. Our findings reveal a strong and consistent correlation between* $CFR$ *and a set of urban parameters, which we uniquely classified into outdoor, indoor, and personal factors. By integrating these variables into a probabilistic model, we demonstrated that it is possible to accurately predict* $CFR$ *across diverse geographic and socio-environmental contexts.*

*The robustness of our model was further validated by its scalability and applicability to other airborne infectious diseases, including influenza and pneumonia, suggesting that the framework is adaptable and broadly relevant for public health risk assessment. Our results underscore the importance of considering urban environmental characteristics such as population density, building configuration, and microclimate conditions when evaluating the risk and severity of airborne disease outbreaks. These insights are particularly valuable for policymakers and public health officials, as they provide a data-driven basis for resource allocation, emergency response planning, and the design of targeted interventions at both local and regional levels. Moreover, our approach advances the field of spatial epidemiology by moving beyond traditional country- or county-level analyses, enabling fine-grained, spatially resolved assessments at the neighborhood and city scale. This level of detail is crucial for identifying vulnerable populations and urban hotspots, thereby supporting more effective and equitable public health strategies.*

*In summary, this work contributes a novel, scalable methodology for quantifying the impact of urban factors on disease severity, reinforcing the need to integrate physical, environmental, and demographic data in epidemic modeling. Our findings highlight the critical role of urban environmental characteristics in shaping public health outcomes and underscore the value of integrating physical urban descriptors into epidemiological risk assessments. This approach provides a robust framework for policymakers and public health officials to identify vulnerable areas and to design targeted interventions that mitigate the impact and severity of airborne infectious diseases in urban environments.*

**Comment #10:** Language and Style: Review the manuscript for clarity and coherence. Ensure that complex concepts are explained concisely and that language is accessible to a broad audience of educators and researchers.

**Response #10:** Manuscript was reviewed for clarity and coherence.

**Comment #11:** Verify that all references are recent, relevant, and properly formatted according to the journal's guidelines. Ensure consistency in citation style throughout the manuscript.

**Response #11:**  The listed references are all relevant and provide the necessary information to support the text of the manuscript and SI. The citation style in our manuscript is that found in recent research papers published in PLOS Digital Heath.

**Comment #12:** Address all reviewer comments comprehensively, particularly those related to methodology clarity, theoretical grounding, and implications for practice.

**Response #12:** To the best of our capability, we have addressed of reviewers’ comments in a comprehensive fashion insisting of the methodology so that the theoretical model at the core of our work is well grounded and understandable by the reader. We did check that all numerical data are correct and accessible to the public to ensure that this work can be reproduced by others.
